# Nucleolus association of chromosomal domains is largely maintained in cellular senescence despite massive nuclear reorganisation

**DOI:** 10.1371/journal.pone.0178821

**Published:** 2017-06-02

**Authors:** Stefan Dillinger, Tobias Straub, Attila Németh

**Affiliations:** 1 Biochemistry Center Regensburg, University of Regensburg, Regensburg, Germany; 2 Biomedical Center, Bioinformatics, Ludwig-Maximilians-University, Munich, Germany; Tulane University Health Sciences Center, UNITED STATES

## Abstract

Mammalian chromosomes are organized in structural and functional domains of 0.1–10 Mb, which are characterized by high self-association frequencies in the nuclear space and different contact probabilities with nuclear sub-compartments. They exhibit distinct chromatin modification patterns, gene expression levels and replication timing. Recently, nucleolus-associated chromosomal domains (NADs) have been discovered, yet their precise genomic organization and dynamics are still largely unknown. Here, we use nucleolus genomics and single-cell experiments to address these questions in human embryonic fibroblasts during replicative senescence. Genome-wide mapping reveals 1,646 NADs in proliferating cells, which cover about 38% of the annotated human genome. They are mainly heterochromatic and correlate with late replicating loci. Using Hi-C data analysis, we show that interactions of NADs dominate interphase chromosome contacts in the 10–50 Mb distance range. Interestingly, only minute changes in nucleolar association are observed upon senescence. These spatial rearrangements in subdomains smaller than 100 kb are accompanied with local transcriptional changes. In contrast, large centromeric and pericentromeric satellite repeat clusters extensively dissociate from nucleoli in senescent cells. Accordingly, H3K9me3-marked heterochromatin gets remodelled at the perinucleolar space as revealed by immunofluorescence analyses. Collectively, this study identifies connections between the nucleolus, 3D genome structure, and cellular aging at the level of interphase chromosome organization.

## Introduction

The spatiotemporal regulation of genomes correlates with transcription, replication, recombination and repair. In the post-genomic era a new model of human genome organization emerged, which is largely based on high-throughput genomics analyses. A key concept of this model is that chromosomal domains, megabase-ranged functional units of the chromosomes, represent an essential operational level of genome regulation (reviewed in [1–8]). However, several questions remained open about the domain organization of human chromosomes. These include the dynamics and functional consequences of chromosomal domain interactions with nuclear sub-compartments in different cell types and under various physiological conditions. The nucleolus is a paramount example for the functional organization of the genome in space and time. Active nucleolar organizer regions (NORs) from different chromosomes build in all cell types a microscopically visible, dynamic nuclear compartment after mitosis, which disassembles again during the next cell division. Consequently, nucleolus-associated chromatin [9] also undergoes cyclic changes in proliferating cells. While the involvement of the nucleolus in ribosome biogenesis, furthermore in facilitating cell cycle progression, stress sensing and RNP function is well characterized [10,11], we just begin to uncover the molecular characteristics of the nucleolus-associated chromatin and to understand the role of the nucleolus in genome organization and function [12–14]. Nucleolus-associated chromosomal domains (NADs) represent the mappable genomic fraction of the nucleolus-associated chromatin. NADs were first identified in HeLa cervical carcinoma and HT1080 fibrosarcoma cells using the combination of high-throughput genomics and immuno-FISH analyses [15,16]. These studies provided us with a snapshot of global genome organization in and around the nucleolus and revealed that NADs represent mainly, but not exclusively a specific heterochromatin compartment. In addition, the mechanistic role of several *cis-* and *trans*-acting factors in chromosomal domain–nucleolus interactions has also been initially addressed [17–20]. However, comprehensive high-resolution maps of NADs and the characterization of the nucleolus-associated genome of normal diploid cells have not been determined. Moreover, the involvement of NADs in the formation of interphase chromosome structure and its dynamics during diverse cellular processes such as cellular aging, differentiation or cell cycle, remained largely unknown.

Cellular senescence is a stable arrest of cell proliferation, whose common form, replicative senescence, is induced by telomere attrition and chromosomal instability. In human and other multicellular organisms, cellular senescence plays a pivotal role in several physiological processes, namely tumour suppression, tissue repair, embryonic development and organismal aging [21,22]. Notably, senescence-related, genome-wide reprogramming of gene expression is accompanied by massive structural reorganization of chromatin [23–28]. Changes in the spatial organization of chromosomes include the formation of senescence-associated distension of satellites (SADS), an early and consistent marker of cellular senescence [29], which can be followed by the development of senescence-associated heterochromatin foci (SAHF) under certain conditions [30,31]. Remarkably, SAHF-like chromosome condensation can be induced also in a senescence-independent manner [32]. The nucleolar hallmarks of senescent cells include increased size and fusion of nucleoli in mammals, furthermore instability of ribosomal DNA (rDNA) in yeast (reviewed in [33,34]). Notably, the instability of the rDNA cluster is considered as a key control element of genome maintenance and inducer of cellular senescence in yeast [35,36]. While recent works highlighted the involvement of various histone modifications, lamins and DNA methylation in shaping the epigenetic landscape during cellular senescence in mammals (reviewed in [37–40]), the molecular details of senescence-related nucleolar remodelling are still elusive.

Here we present high-resolution NAD maps of young and replicative senescent IMR90 human embryonic fibroblasts. Our findings identify a central role for the nucleolus in the nuclear arrangement of specific transcriptionally silent chromosomal domains in proliferating and senescent cells, and suggest molecular mechanisms involved in the regulation of nucleolus-associated chromatin and spatial genome organization.

## Results

### Mapping of NADs in primary human cells

**Fig 1:**
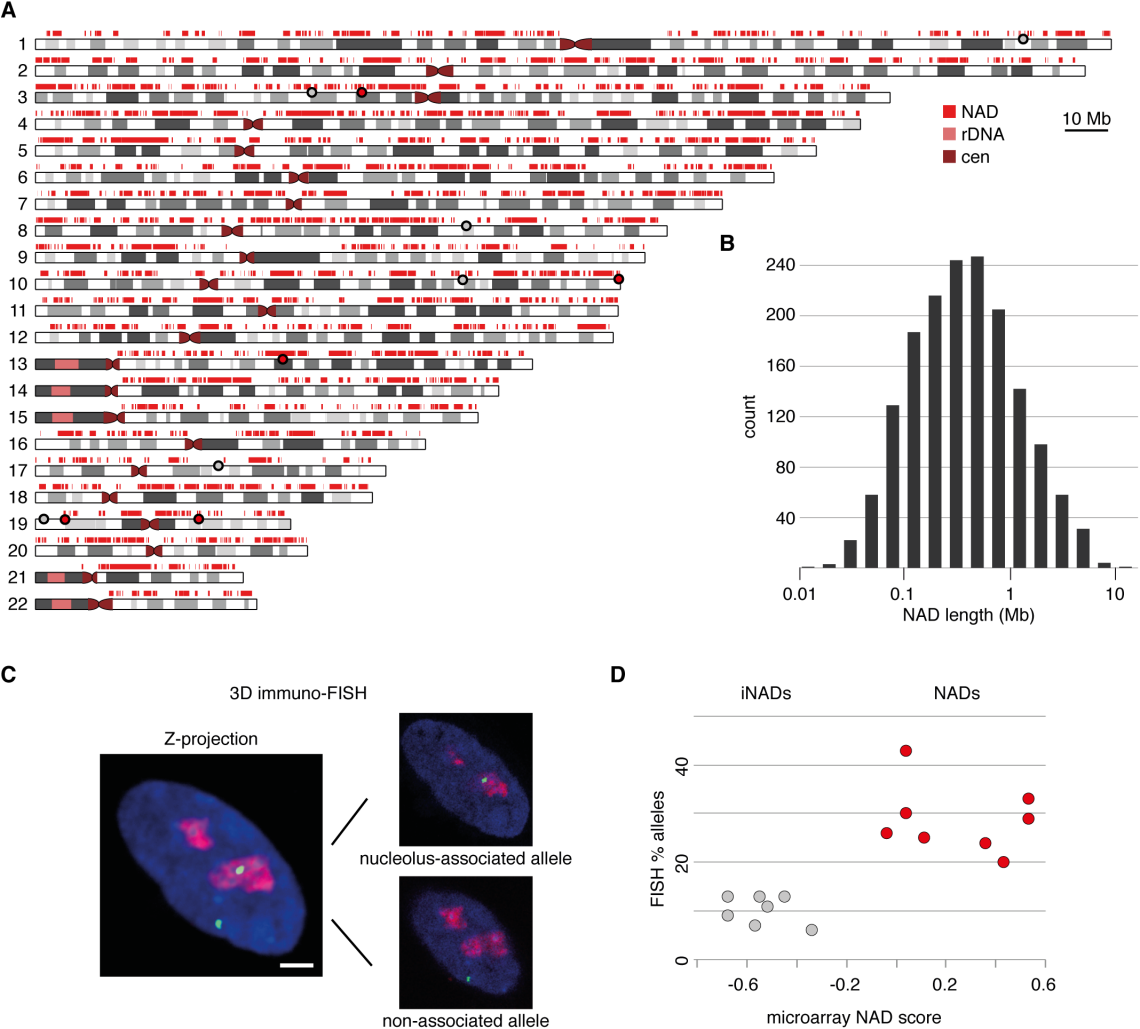
Map and genome features of nucleolus-associated chromosomal domains (NADs) in IMR90 primary human embryonic fibroblast cells. (**A**) Distribution of NADs along human autosomes. NADs are indicated by red rectangles over the ideograms of the chromosomes. Note that the p-arms of the five acrocentric chromosomes (13, 14, 15, 21 and 22), centromeres and some pericentromeric regions were not analysed because they are not present in the current human genome assembly. (**B**) Histogram of NAD sizes. Median = 361kb, a total of 1,646 NADs were identified. (**C**) 3D immuno-FISH analysis of NAD and inter-NAD regions (iNADs) in IMR90 cells. Nucleolus association of a chromosomal domain is illustrated by showing the Z-projection of an IMR90 nucleus on the left and the corresponding single light optical sections with the associated and non-associated allele on the right. BAC hybridization signals are shown in green, nucleolar staining in red and DAPI counterstain in blue (scale bar: 1.6 μm). (**D**) Hybridization signals (percentage of nucleolus-associated alleles) are plotted against the according microarray signals (average log2-fold difference of the nucleolar signal over the background). Red and grey circles indicate genomic regions that reside in NADs and iNADs, respectively (see S2 Table for further details). The positions of the BAC clones used in 3D immuno-FISH experiments to monitor NADs and iNADs are shown also in (**A**) by red and grey circles, respectively.

In two independent experiments, nucleoli of each young and proliferating IMR90 fibroblasts were isolated by biophysical disruption of cells and subsequent fractionation by differential centrifugation. To assess the quality of the purification, the enrichment of the nucleolar transcription factor UBTF and ribosomal DNA (rDNA) were monitored in immunoblot and quantitative PCR experiments, respectively, and the nucleolar fraction was examined also by microscopy (S1 Fig). The DNA of isolated nucleoli was extracted and subjected to comparative genomic hybridization experiments on whole genome tiling microarrays to identify the non-repetitive DNA content of the nucleolus-associated genome and to address its chromosomal organization. The bimodal nature of hybridization signals provided the basis for genome-wide mapping of NADs with the use of a two-state hidden Markov model (HMM) analysis. By using this approach 1,646 autosomal NADs of young, proliferating IMR90 cells were discovered (Fig 1A, S2 Fig, and S1 Table). We refer here to this comprehensive list of IMR90 NADs simply as ‘NADs’, and to the previously identified HeLa NADs [15], which were mapped using an 85%-threshold-based method, as ‘top NADs’. NADs cover 1.2 billion bp, approximately 38% of the annotated human genome, and their median sequence length (361 kb) resides in the typical size range of the higher order structural and functional chromosomal domains of mammalian genomes (Fig 1B).

In order to validate the nucleolus association of selected chromosomal regions, 3D immuno-FISH experiments were performed. Nucleoli were labelled by immunofluorescence staining of nucleophosmin (NPM1) and different genomic regions by fluorescence hybridization. The frequency of nucleolus interaction was determined for several genomic regions and plotted against the NAD score value, which was calculated from the microarray data by averaging the log-enrichment values of two replicate experiments. Additionally, our previously collected 3D immuno-FISH data [15] was also integrated in the analysis (Fig 1C and 1D, and S2 Table). The results of population-based and single-cell analyses correlate well, as chromosomal regions with high NAD scores showed typically more frequent association with nucleoli in 3D immuno-FISH than regions with low NAD scores.

### Comparative epigenomics of NADs and inter-NAD regions (iNADs) reveals specific heterochromatic features of NADs

**Fig 2:**
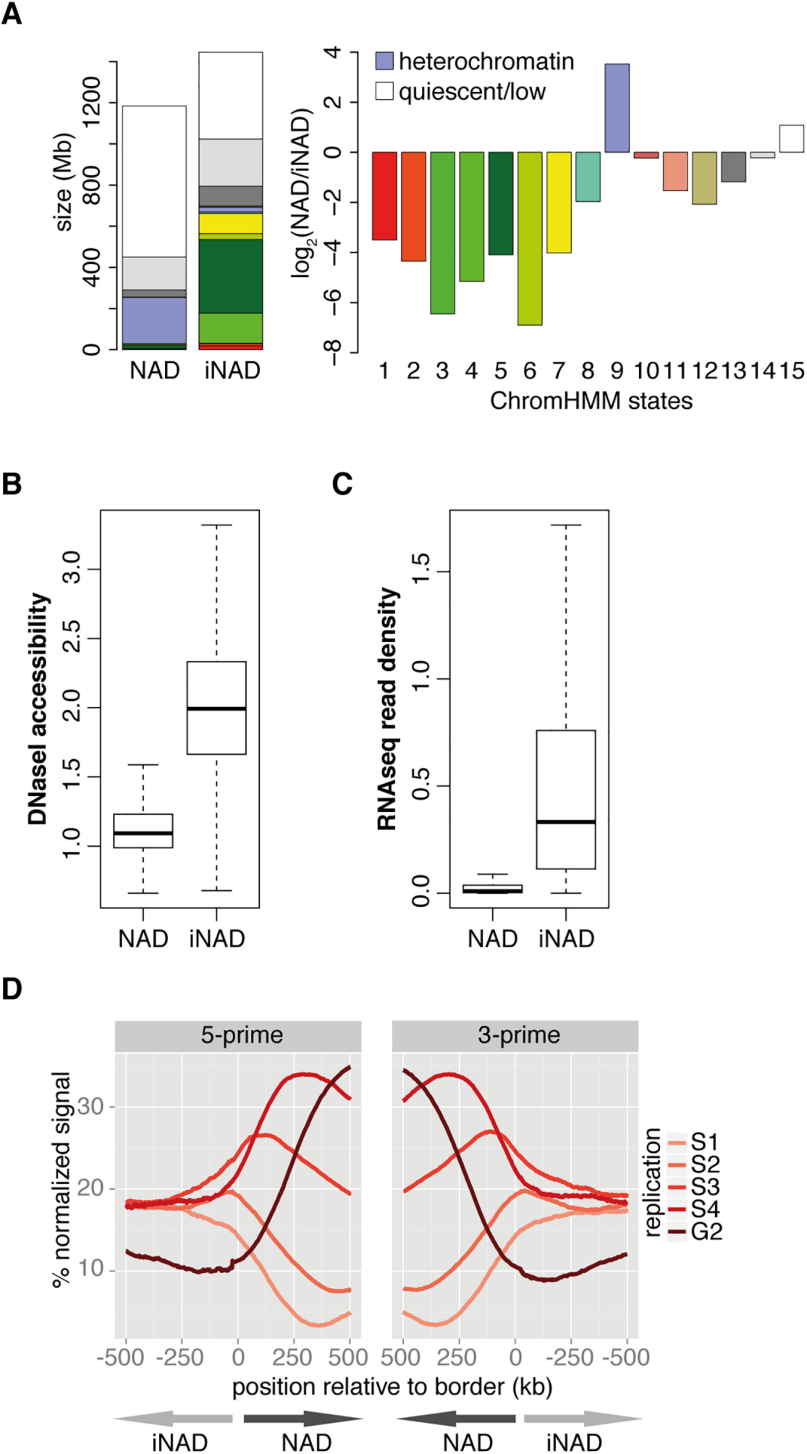
Comparative epigenomics of NADs and inter-NAD regions (iNADs) reveals specific heterochromatic features of NADs. (**A**) Distribution of different chromatin states in NADs and iNADs. Bar graphs show total and relative amounts of ChromHMM states in NADs and iNADs on the left and right, respectively. The chromatin states and their colour code correspond to the Primary Core Marks segmentation 15-state ChromHMM model of the Roadmap Epigenomics Project [41]. Chromatin states specifically enriched in NADs are indicated. Red: Active Transcriptional Start Site (TSS), OrangeRed: Flanking Active TSS, LimeGreen: Transcription at gene ends, Green: Strong transcription, DarkGreen: Weak transcription, GreenYellow: Genic enhancers (Enh), Yellow: Enh, MediumAquamarine: ZNF genes & repeats, PaleTurquoise: Heterochromatin, IndianRed: Bivalent/Poised TSS, DarkSalmon: Flanking Bivalent TSS/Enh, DarkKhaki: Bivalent Enh, Silver: Repressed PolyComb, Gainsboro: Weak Repressed PolyComb, White: Quiescent/Low. (**B**) Boxplot of DNaseI accessibility in NADs (n = 1646) and iNADs (n = 1669). The average accessibility per base and segment were calculated from GSM468792. (**C**) Boxplot of RNA-seq read densities. For each NAD (n = 1646) and iNAD (n = 1669) the average number of reads per base in GSM438363 were calculated. (**D**) Replication timing profiles around NAD borders. Average percentage-normalized signals of different replication domains within a distance of 500 kb from aligned 5’ and 3’ NAD borders are shown. Repli-Seq signals from five different time points of the cell cycle (S1-S4 and G2) were taken from Pope *et al*.,[42] and averaged over 1 kb windows. Only NADs with a width >500 kb were considered (n = 652). The arrows drawn at the bottom show away from the aligned NAD/iNAD borders.

In order to uncover the chromatin features of NADs at the genome level, the 15-state chromatin HMM (ChromHMM) map of IMR90 cells [41] was quantitatively analysed. The size of NAD and iNAD genomic regions occupied by the various chromatin states was identified and illustrated on a bar graph. Next, the values of each chromatin state were calculated for the entire population of NADs and iNADs, and their log2 ratios were plotted (Fig 2A). The results provide compelling evidence that NADs are depleted in active chromatin features and they can be mainly characterized by the ‘heterochromatin’ and ‘quiescent/low’ ChromHMM states. The main features of these chromatin states are high levels of DNA methylation, low DNaseI accessibility, and low incidence of genes, which are mainly repressed. Further investigations of the overall DNaseI accessibility and transcriptional activity in NADs and iNADs revealed large differences with clearly lower values in NADs (Fig 2B and 2C), supporting the results of the ChromHMM analysis.

To identify the replication timing profile around NAD borders, NADs were aligned at their 5’ and 3’ ends (p to q chromosome orientation), and normalized Repli-seq signals were averaged in 500 kb windows spanning the borders. Replication signals were measured recently at five different time points of the S/G2 phases of the cell cycle [42], and their distributions appear as five waves around NAD borders (Fig 2D). The waves show a regular spatiotemporal pattern within NADs, in which early-to-late replication timing correlates with the distance to the border. Interestingly, only the depletion of the latest replicating fraction (G2) is notable outside of NADs in the 0 to -500kb distance range, whereas the signal intensities of the other four fractions (S1 to S4) get indistinguishable with increasing distance. Altogether, the result is in good agreement with the late-replicating nature of heterochromatin, the characteristic chromatin state of NADs.

### Comparative genomics of NADs, iNADs, lamina-associated domains (LADs) and inter-LADs (iLADs) uncovers NAD-specific genomic features

**Fig 3:**
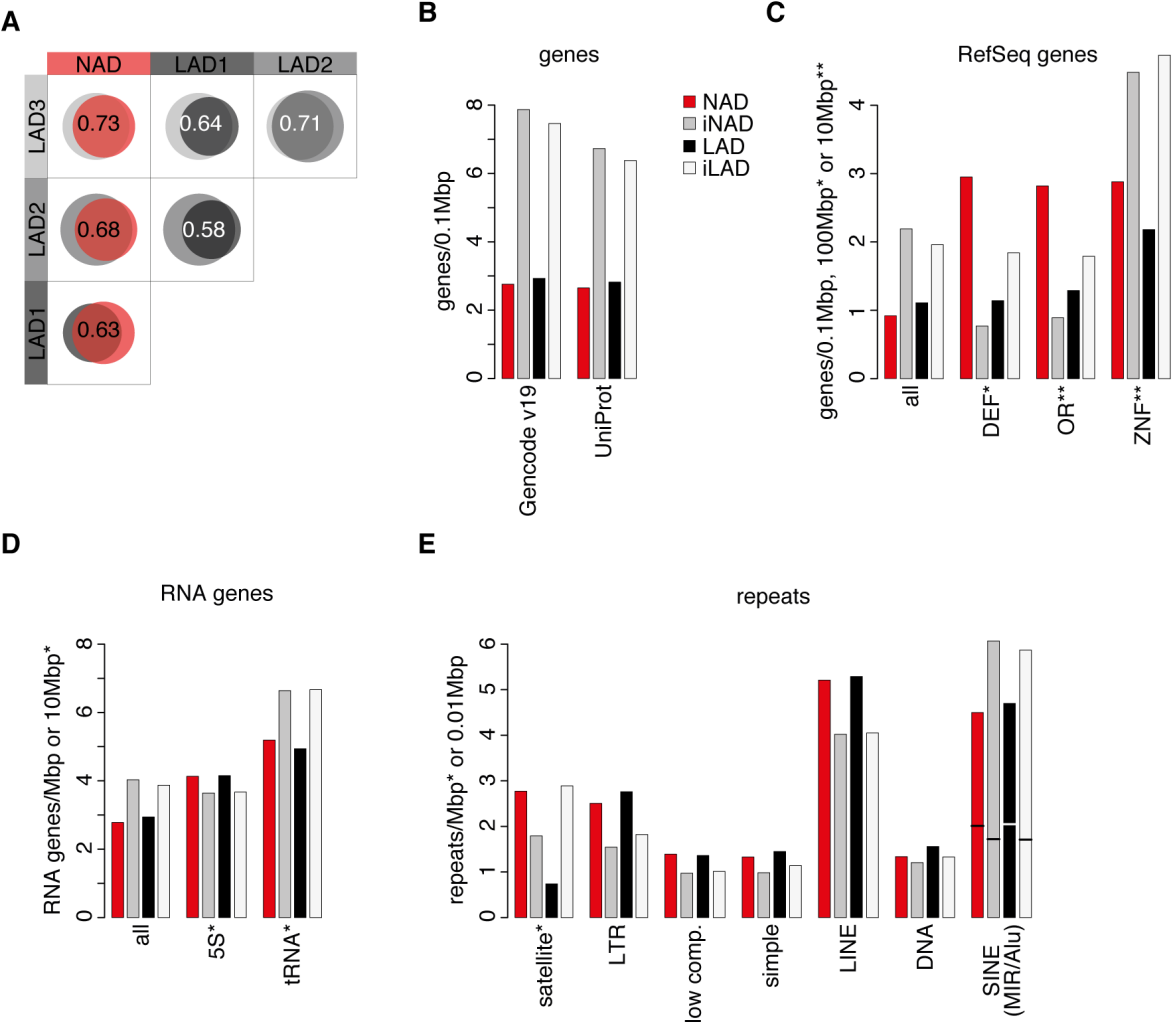
Comparative genomics of NADs, iNADs, lamina-associated domains (LADs) and inter-LADs (iLADs) uncovers NAD-specific genomic features. (**A**) Venn diagrams and Jaccard coefficients show the extent of overlap between NADs and LADs. LAD1: LADs of Tig3 cells [44], LAD2 and LAD3: LADs of IMR90 cells [24,25]. (**B**) Bar graphs show Gencode v19 and UniProt gene frequencies in NADs (red), iNADs (grey), LADs (black), and iLADs (white) based on UCSC Table Browser data. (**C**) RefSeq gene (ZNF, OR and DEF indicate zinc finger, olfactory receptor and defensin gene families, respectively) frequencies in NADs, iNADs, LADs, and iLADs. (**D**) Non-coding RNA gene (‘RNA genes’) and (**E**) repeat frequencies in NADs, iNADs, LADs, and iLADs. The SINE repeat bars are divided with a horizontal line into MIR (bottom) and Alu (top) sub-groups.

As described above, several lines of experimental evidence point to a considerable enrichment of heterochromatin in NADs. The constitutive heterochromatin of cultured mammalian cells predominantly localizes to the nuclear periphery, pericentromeric bodies and the perinucleolar region, and these nuclear sub-compartments are to large extent functionally overlapping (recently reviewed in [43]). Genomic analyses of the nuclear periphery identified the maps of lamina-associated domains (LADs) in proliferating Tig3 human fibroblasts by using the DamID method [44], and later also in IMR90 cells by a Lamin B1 ChIP-seq approach [24,25]. To statistically evaluate the similarity of LADs and NADs of human embryonic fibroblasts, the three LAD datasets were compared to the NAD list of IMR90 cells and to each other in a quantitative manner. As illustrated by the large intersections of the Venn diagrams, the IMR90 NADs show a substantial overlap with the LADs of human diploid fibroblasts (Fig 3A). However, about one-third of the total NAD- and LAD-covered genomic regions are non-overlapping and chromosome-specific differences in the patterns are clearly recognizable (S3 Fig). To identify differences between genomic features of NADs and inter-NAD regions (iNADs), furthermore LADs and inter-LADs (iLADs) of the annotated human genome iNADs, the incidence of various sequence features was addressed in these chromosomal domains of human primary fibroblasts (Fig 3B–3E). The results revealed that defensin (DEF) and olfactory receptor (OR) genes are enriched in otherwise gene-poor NADs, whereas they are depleted in gene-rich iNADs and show intermediate levels in LADs and iLADs. Although zinc finger (ZNF) genes are depleted in NADs, the extent of their depletion is less than the overall depletion of protein-coding genes in NADs. A similar ZNF gene distribution can be observed between LADs and iLADs (Fig 3C). Non-coding RNA genes are also less prevalent in NADs and LADs than in iNADs and iLADs, however 5S RNA genes are slightly enriched in NADs and LADs (Fig 3D). Repetitive DNA analyses showed that satellite repeats and LTR elements are enriched in NADs, and to a far lesser extent also low complexity repeats, simple repeats, LINEs and DNA repeats. In contrast, SINEs are depleted in NADs due to the lower incidence of Alu repeats in these genomic regions. Similar distributions of repetitive elements can be observed between LADs and iLADs with the clear exception of satellite repeats, which are more frequent in iLADs than in LADs (Fig 3E). Taken together, satellite repeats, furthermore OR and DEF gene-coding regions represent the most specific, characteristic genomic elements of NADs. While the prevalence of specific sequence features in NADs is similar to that of top NADs [15], the enrichment of individual features is less pronounced.

### Weak nucleolus association is characteristic for escaper regions on the X chromosome

**Fig 4:**
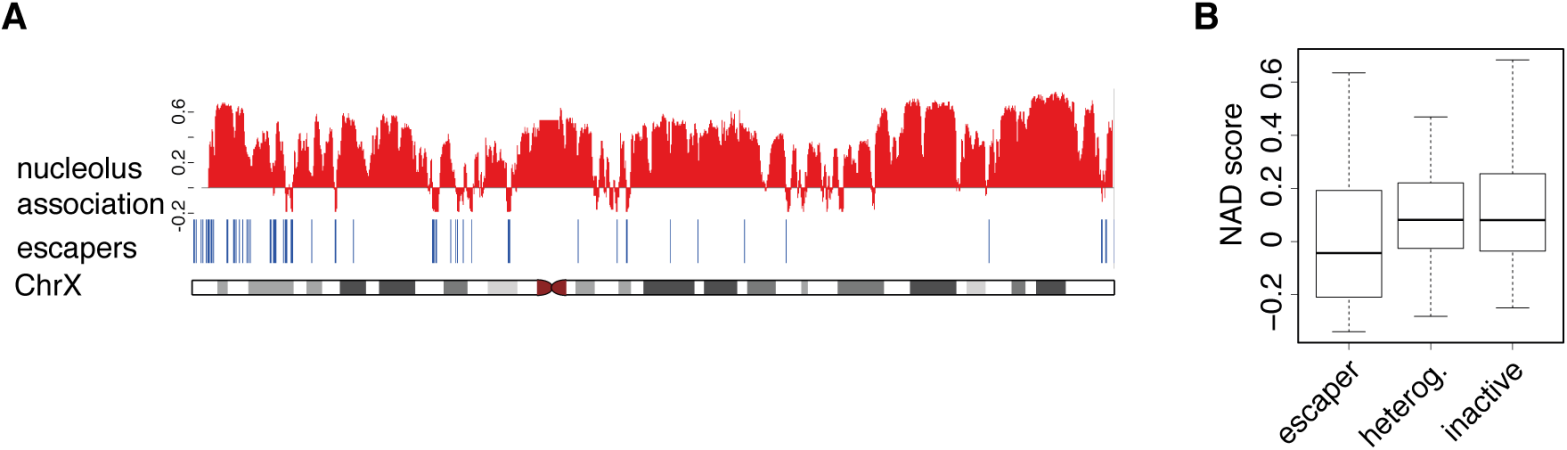
Weak nucleolus association is characteristic for escaper regions on the X chromosome. (**A**) Microarray signals of nucleolus-association (log2-fold difference of the nucleolar signal over the background, combined from two biological replicate experiments) and X escaper gene positions are shown on the top of the X chromosome ideogram. (**B**) Boxplot of NAD scores in ‘escaper’, ‘heterogeneous’ and ‘inactive’ genes (classification based on Carrel and Willard [45]).

As IMR90 cells possess one active and one inactive X chromosome having substantially different chromatin conformations and no Y chromosome, the sex chromosomes were not included in the aforementioned genome-scale NAD analyses. In an extended mapping of NADs a few regions of the Y chromosome emerged as nucleolus associated, due to cross-hybridization or false assignment on the microarrays. In contrast, almost the entire X chromosome appeared as NAD in this analysis. The heterochromatic nature of the inactive X chromosome and the frequent association of the entire chromosome with the perinucleolar heterochromatin might explain that irrespective from the sub-nuclear localization of the active X, most regions of the X chromosome are classified as NAD (S4 Fig). Nevertheless, certain regions of the X chromosome were identified as iNADs. Since some of them clearly overlapped with the pseudo-autosomal regions that escape X inactivation (Fig 4A), the correlation between escaper genes and iNADs of the X chromosome was further addressed. The ‘escaper’, ‘heterogeneous’ and ‘inactive’ states of genes were classified according to Carrel and Willard ([45] and S3 Table) and NAD score values were computed for all three groups. A boxplot representation of the results demonstrates that the escaper group is less frequently associated with nucleoli than the two others (Fig 4B). The visualization of microarray data along with escaper gene positions provides a more detailed picture. Besides the fact that nearly all iNAD regions contain escaper genes, the remaining escapers also show a strong coincidence with weaker nucleolus association within NADs (Fig 4A). These observations strongly suggest that local spatial distension, for instance looping of active genes leads to their efficient separation from the more compact heterochromatin of the inactive X chromosome during nucleolus isolation.

### Integration of NAD and Hi-C data reveals that NAD-NAD interactions dominate in the 10–50 Mb distance range over iNAD-iNAD and heterotypic NAD-iNAD interactions

**Fig 5:**
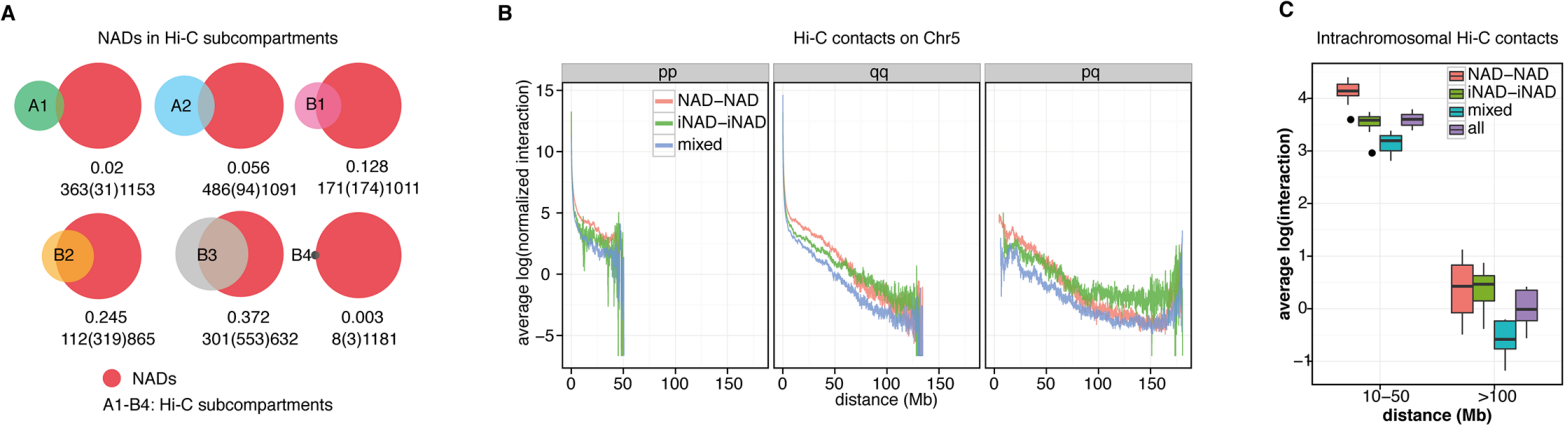
Integration of NAD and Hi-C data reveals that NAD-NAD interactions dominate in the 10–50 Mb distance range over iNAD-iNAD and heterotypic NAD-iNAD interactions. (**A**) Venn diagrams of NADs and A1-A2, B1-B4 Hi-C subcompartments show heterochromatic features of NADs. Jaccard coefficients and the sizes of overlapping and non-overlapping regions (in Mb) are shown below the diagrams. (**B**) Average intrachromosomal NAD-NAD, iNAD-iNAD and NAD-iNAD interactions at different distances on chromosome 5. (**C**) Boxplots of genome-wide NAD-NAD, iNAD-iNAD and NAD-iNAD interaction frequencies measured at 10-50Mb and >100Mb distances. Data points are the average interaction values of 10 kb windows matching a specific interaction class (NAD-NAD, iNAD-iNAD, mixed) and calculated for chromosomes 1 to 12 (n = 12).

Chromosome Conformation Capture (3C) provides a way to divide chromosomes into domains by measuring contact probabilities between chromosomal segments. Hi-C analyses with increasing resolution led to the genome-wide determination of A and B compartments [46], topological domains [47], and finally contact domains [48] of human chromosomes. However, this methodology does not deliver information about the nuclear position of chromosomal domains and their interaction with nuclear bodies. To integrate complementary information about the spatial organization of the human genome in IMR90 cells, a comparative genomic analysis was performed using the NAD data and the to date highest resolution Hi-C datasets of this cell line [48]. Venn diagrams show the overlap between NADs and the six genomic subcompartments, which were determined by Hi-C-based segregation of the human genome (Fig 5A). First, the results demonstrate that there is only little overlap between NADs and the euchromatic, gene dense A1-A2 subcompartments. Second, nearly half of the B1 subcompartment, which represents facultative heterochromatin, coincides with NADs. Third, approximately three-quarter of the B2 and two-third of the B3 subcompartments, primarily constitutive heterochromatin, correspond to NADs. Importantly, these are the largest Hi-C subcompartments. Of note the strongest overlap between B2 and the NADs, which was also detected with the top NADs [48], and also the increased B3 –NAD overlap. Finally, NADs coincide to a greater extent with B4 than with A subcompartments. The interpretation of this overlap is ambiguous due to the minute size of B4. B4 contains KRAB-ZNF gene clusters and displays a specific chromatin pattern, and it is the far smallest Hi-C subcompartment. Altogether, the results of the comparative analysis of Hi-C subcompartments and NADs are largely consistent with the results of the genomic analyses shown in Figs 2 and 3, and uncover that 74% of NADs reside in B2/B3-type constitutive heterochromatic chromosomal regions.

In the nucleus of mammalian cells the chromosomes occupy distinct territories [1,49,50], and their ultrastructural organization is influenced by intra-chromosomal association of chromosomal domains. To compare the contribution of NADs and iNADs to intra-chromosomal interactions at different size scales, frequencies of NAD-NAD, iNAD-iNAD and mixed NAD-iNAD contacts were calculated for each chromosome from IMR90 Hi-C datasets and plotted against linear sequence distance (Fig 5B and S5 Fig). Intra- and interarm interactions were visualized separately to consider a possible influence of centromeres on intrachromosomal contact probabilities. Visual inspection of the plots revealed that NAD-NAD contacts dominate at the 10–50 Mb distance range over iNAD-iNAD and mixed contacts. Over larger distances iNAD-iNAD contacts became the most frequent, whereas NAD-iNAD contact probabilities are clearly the lowest ones at all distance ranges. Moreover, a strong increase of contact frequencies can be observed in the <10 Mb distance range, which corresponds well to the size range of topologically associating domains (TADs). TADs are considered as the basic units of chromosome folding and they can be defined by measuring genomic interaction frequency changes along the chromosomes: the interaction frequencies of two loci are high within TADs but sharply drop at the boundary between neighbour TADs [51]. In order to visualize NAD-NAD and iNAD-iNAD contacts at >10Mb distances in a locus-specific manner, the Hi-C contact frequencies of individual chromosomes were displayed also on heat maps (S6 Fig). To aid the identification of homotypic contacts, NADs or iNADs were masked in additional individual heat maps. Strikingly, NAD/iNAD and Hi-C-contact-based TAD segmentations of the chromosomes revealed highly similar patterns. In addition, the heat maps showed again that NAD-NAD contacts are enriched at 10–50 Mb and depleted at >50 Mb distances.

Next we calculated the average interaction intensity within each NAD-NAD, iNAD-iNAD, and NAD-iNAD contact to address the involvement of NADs in chromosomal contacts at different distance ranges. To exclude intra-NAD contacts from the analyses, contact frequencies at <10Mb distances were omitted. The contact frequencies of different contact classes were calculated at two different distance ranges, at 10-50Mb versus >100Mb and shown in a boxplot diagram (Fig 5C). Due to the <100Mb size of human chromosomes 16 to 22 and the fact that the p arms of the acrocentric chromosomes 13 to 15 are not annotated, only chromosomes 1 to 12 were investigated here. The boxplots show that homotypic interactions of chromosomal domains are the prominent ones in the organization of chromosome territories at >10Mb distances. Importantly, the results also reveal that NAD-NAD contacts are the dominant ones at the 10-50Mb distance range. Next, the contribution of the different contact classes to the overall contact frequencies within and between chromosome arms was visualized (S7 Fig). It is clearly recognizable that NAD-NAD interactions are the dominant ones within intraarm contacts over 10Mb distances, whereas NAD-NAD and iNAD-iNAD interactions show similar frequencies within interarm contacts. The prevalence of NAD-NAD interactions in the intraarm contacts compared to interarm contacts suggests that the centromere may reduce interarm contact probabilities, however the linear distance seems to play the major role in determining intrachromosomal NAD-NAD contact probabilities.

### Replicative senescence causes fusion of nucleoli, but only local, transcription-dependent changes in NADs

**Fig 6:**
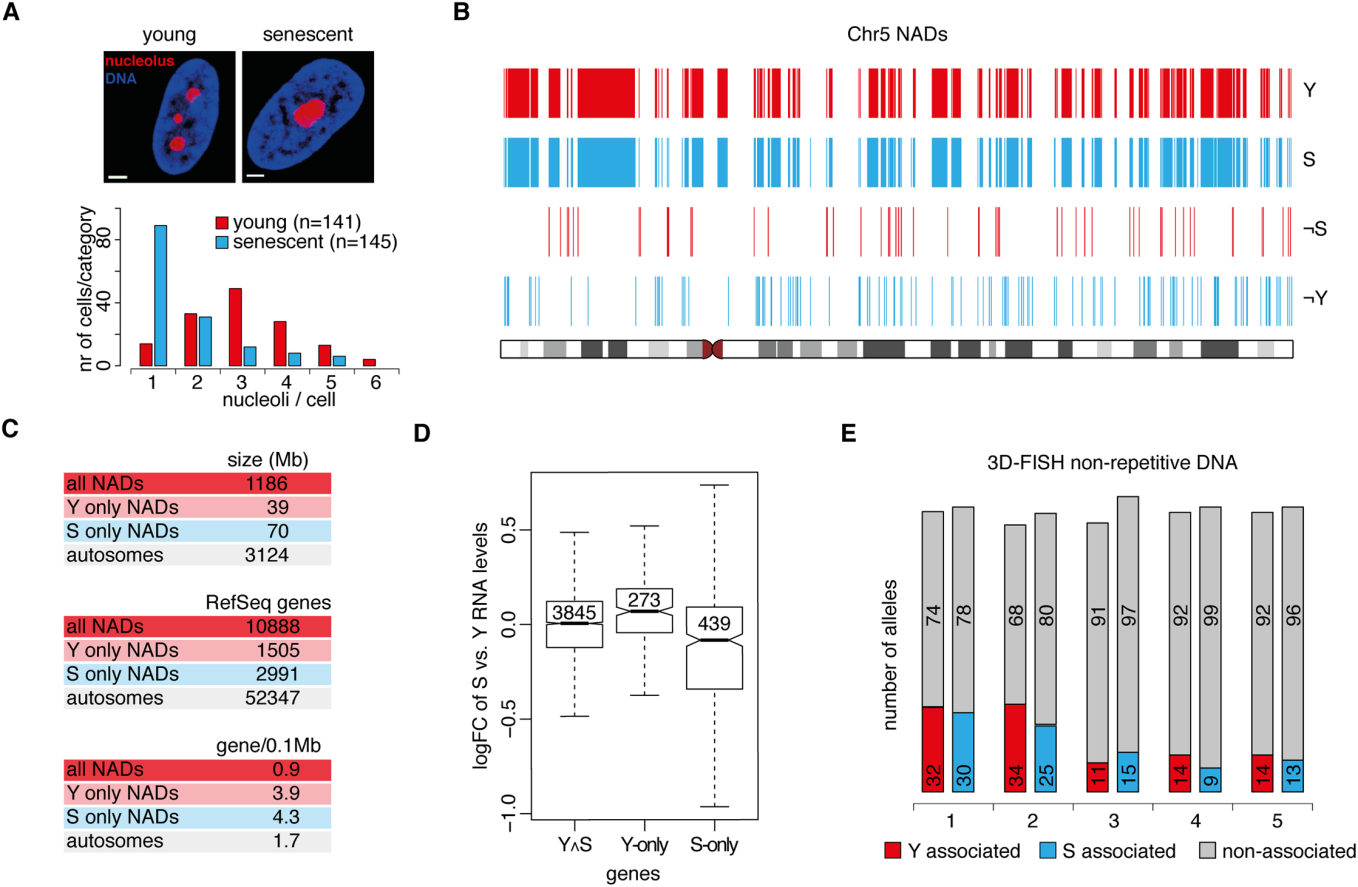
Replicative senescence causes fusion of nucleoli, but only local, transcription-dependent changes in NADs. (**A**) Bar graph of nucleolus number in young, proliferating (‘Y’) and senescent (‘S’) IMR90 cells. Proliferating cells have 3.0±1.2 and senescent cells 1.7±1.1 nucleoli per nucleus. Z projections of mid-sections of representative confocal microscopy images are shown on the top. Nucleolar staining is shown in red and DAPI counterstain in blue (scale bars: 1.6 μm). (**B**) Maps of NADs on chromosome 5 from young and senescent cells. Genomic regions associated with nucleoli only in young (¬S) or senescent (¬Y) cells are shown also as individual tracks. (**C**) Y-only and S-only NADs are enriched in protein-coding genes compared to all NADs and the genome. RefSeq gene data were obtained from the UCSC Table Browser. (**D**) Boxplots show positive correlation of senescence-related loss of nucleolus association and gene activation. Global gene expression changes (log2 fold change in senescent versus young cells) in constitutive (Y∧S), Y-only and S-only NAD genes are shown. The notches are defined as +/-1.58*IQR/sqrt(n) and represent the 95% confidence interval for each median. Group means are significantly different for all comparisons (p-value < 0.05, Tukey HSD). (**E**) Stacked columns show that the association frequency of five selected genomic regions is similar in young and senescent cells. Nucleolus-association data were collected from 50 cells for each category. BAC clones 1 to 5: RP11-44B13, RP11-173M10, RP11-828F4, RP11-125O21, RP11-81M8.

Next, we addressed the questions how nucleolar morphology, NAD organization and global gene expression alter during cellular aging. IMR90 fibroblasts were grown under standard cell culture conditions and the aging of the cell population was monitored (S8 Fig). In order to evaluate senescence-associated changes in nucleolar number and morphology, proliferating and senescent IMR90 cells were stained for NPM1 by immunofluorescence. Counting of NPM1 signals showed that proliferating cells exhibit typically 2–4 nucleoli and senescent cells a single, large nucleolus (Fig 6A).

In order to discover genome-wide changes of nucleolus-associated chromatin at high resolution, NADs of senescent cells were mapped and compared to NADs of young, proliferating cells. Surprisingly, the two NAD maps were highly similar. Altogether 39 Mb sequence was specific for young NADs and 70 Mb for senescent NADs, which respectively represent 3.3% and 5.9% of the total NAD size (1.2 billion bp) determined in young IMR90 fibroblasts (S4 Table). Importantly, chromosomal fragments that can be determined as young-only or senescent-only NAD regions have a median size of less than 20 kb. Accordingly, most of the changes involved just parts of individual NADs (Fig 6B and S9 Fig). Systematic analysis of sequence features in young-only and senescent-only NAD regions revealed that they are particularly enriched in protein-coding genes (Fig 6C and S4 Table). To identify whether the changes in nucleolus association correlate with transcriptional dynamics gene expression microarray experiments were performed. A targeted bioinformatics analysis revealed that the loss of nucleolus association correlates with an overall significant increase in transcript levels, while the gain of nucleolus association with a significant decrease of it (Fig 6D). Additionally, hierarchical clustering analysis of gene expression microarrays was performed using the data from this and two other recent studies [25,52], in which the same experimental system was investigated. The result showed co-clustering of young and senescent samples providing an additional, robust quality control of the proliferating and senescent states (S10 Fig).

### Nucleolus—satellite repeat cluster interactions are impaired in senescent cells, whereas rDNA and telomeres do not display remarkable changes in nucleolus association frequency

**Fig 7:**
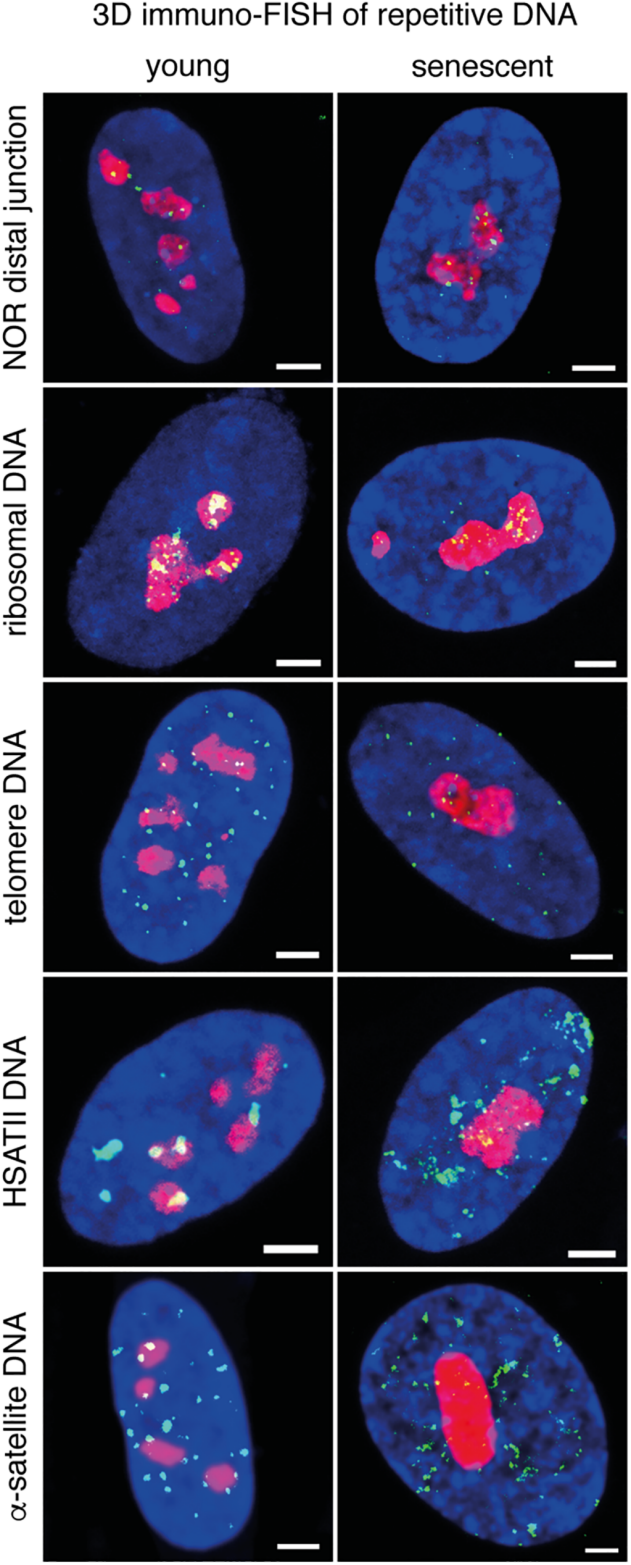
Nucleolus—satellite repeat cluster interactions are impaired in senescent cells, whereas rDNA and telomeres do not display remarkable changes in nucleolus association frequency. Maximal intensity projections of Z-sections of representative confocal microscopy images are shown to aid the visualization of all repeat clusters within a single nucleus. Nucleolar staining is in red, DAPI counterstain in blue and FISH signals are in green on all images (scale bars: 1.6 μm). From top to bottom: The distal junction regions of NORs and rDNA repeat clusters remain associated with nucleoli in senescent cells. The signal intensity of telomeres is reduced in senescence, but their overall association with nucleoli does not show specific changes. HSATII and alpha-satellite repeat clusters display senescence-associated distension and reduced nucleolus association.

To uncover the role of cellular aging on the nuclear position of selected genomic loci 3D immuno-FISH analyses were performed. The comparison of the association frequencies of five chromosomal loci with nucleoli showed only little differences between young and senescent cells (Fig 7). This result is in agreement with the largely unaltered maps of NADs in senescence. In the next experiments the spatial dynamics of tandem repeat arrays was addressed, which build the core of the nucleolus-associated chromatin, but are not present on NAD maps because they reside in non-annotated genomic regions. The clusters of rDNA and the distal junctions of NORs displayed basically no difference between young and senescent cells. There was no indication for complete inactivation of NORs, that is, complete separation of strong hybridization signals from the nucleolus. The distal junctions appeared as discrete loci at the nucleolar periphery, whereas rDNA signals were distributed within the nucleoli. Similar to these loci, the global distribution of telomeres did not show detectable aging-related difference at the nucleolar and peri-nucleolar space, however the signal intensities were severely reduced in replicative senescent cells indicating telomere attrition. In contrast, senescence-associated distension of satellite repeats led to their substantial disassociation from nucleoli (Fig 7). Collectively, the 3D immuno-FISH and genomics analyses showed that mainly satellite repeat arrays and individual genes within NADs are the subjects of nucleolus-associated chromatin reorganization in senescence.

### The senescence-dependent spatial reorganization of H3K9me3-marked chromatin differs at the perinucleolar space and the nuclear periphery

**Fig 8:**
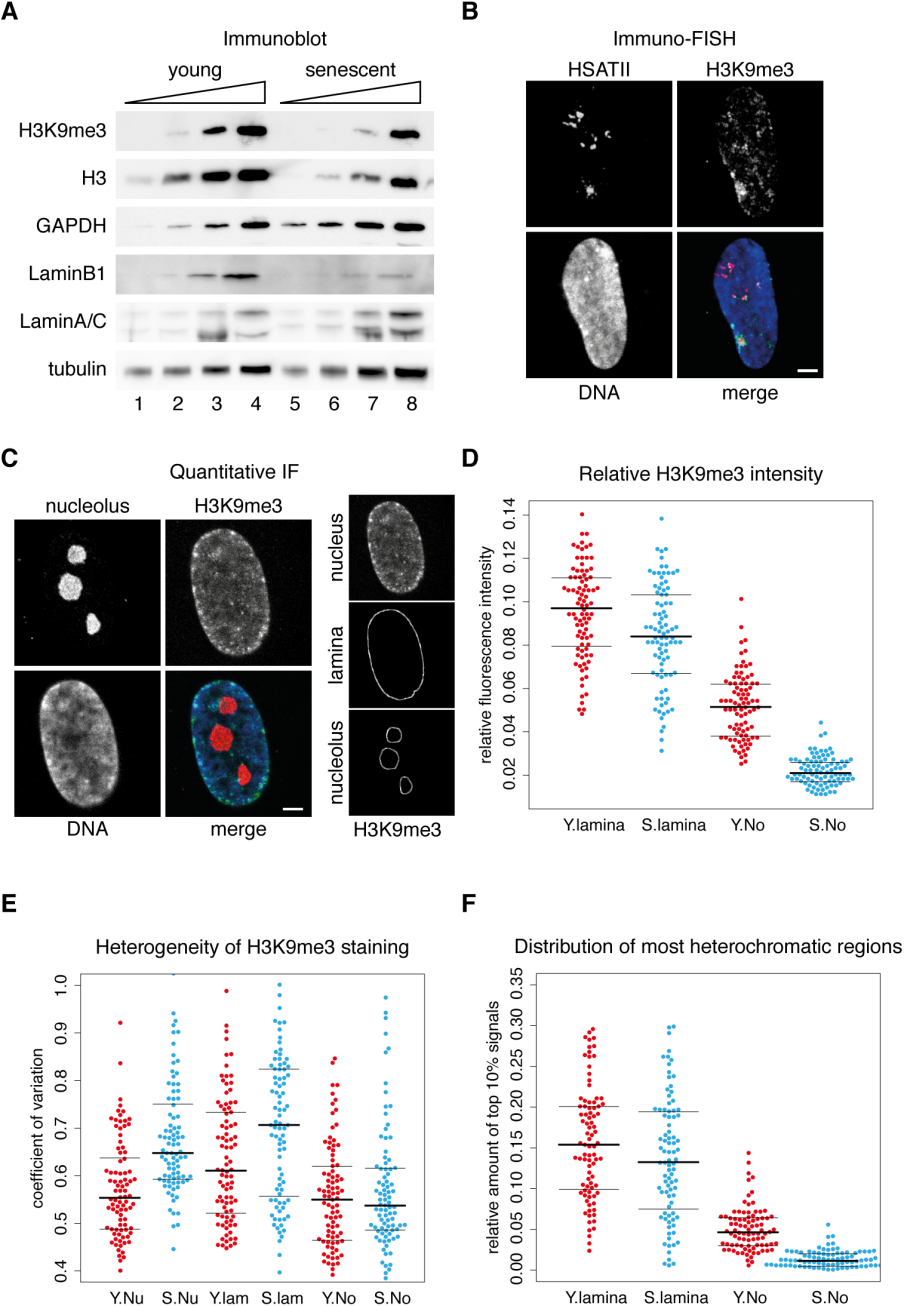
The senescence-dependent spatial reorganization of H3K9me3-marked chromatin differs at the perinucleolar space and the nuclear periphery. (**A**) Semi-quantitative immunoblots show severely decreased H3K9me3 and Lamin B1 levels, less strongly decreased H3 levels and no detectable alterations in Lamin A/C, tubulin and GAPDH levels in senescence. The same amounts of whole cell extracts of young and senescent cells were loaded as serial two-fold dilutions on SDS-PA gels and analysed on immunoblots (see the entire dataset from three independent experiments in S11 Fig). (**B**) H3K9me3 is accumulated at spatially compact satellite repeat clusters. 3D immuno-FISH shows strong co-localization of H3K9me3 and HSATII staining. Mid-section of a representative confocal microscopy image is shown. HSATII FISH signals are in red, DAPI counterstain in blue, and H3K9me3 immunofluorescence signals are in green (scale bar: 1.6 μm). (**C**) Quantitative immunofluorescence analysis of H3K9me3 distribution. The areas of interest are illustrated on a light optical section of a representative confocal microscopy image. The lamina- and nucleolus-associated areas label 240 nm distances from the edges of the DAPI and nucleolus staining, respectively. (**D**) Bee swarm plots of relative fluorescence intensities show senescence-dependent small decrease in H3K9me3 levels at the nuclear periphery and strong reduction at the perinucleolar space. Proliferating and senescent IMR90 cells were stained for H3K9me3 and the relative immunofluorescence intensities were measured at the nuclear periphery (lamina) and at the perinucleolar space (No). Values measured in proliferating cells (‘Y’) are shown in red, values measured in senescent cells (‘S’) are shown in blue. Results from individual cells are illustrated as single data points (n_Y_ = 88, n_S_ = 88). A solid line indicates the median, and thin lines the upper and lower quartile. Median: Y.lamina = 0.095, S.lamina = 0.084; Y.No = 0.052, S.No = 0.022. (**E**) Bee swarm plots indicate more heterogeneous H3K9me3 staining in the nucleus and at the nuclear periphery of senescent cells, but no change in the perinucleolar space. The heterogeneity of staining was calculated as coefficient of variation (C.V. = standard deviation/mean of fluorescence intensity) for the total nucleus (Nu), the nuclear periphery (lamina) and the perinucleolar space (No). Plot labels are as in (**D**). Median: Y.Nu = 0.573, S.Nu = 0.677; Y.lamina = 0.632, S.lamina = 0.709; Y.No = 0.576, S.No = 0.555, n_Y_ = 88, n_S_ = 88. (**F**) Bee swarm plots illustrate robust rearrangement of the most heterochromatic regions in the perinucleolar space. The distribution of the 10% brightest pixels was quantified at the nuclear periphery (lamina) and the perinucleolar space (No). Ratios were calculated compared to the whole nucleus. Plot labels are as in (**D**). Median: Y.lamina = 0.157, S.lamina = 0.137; Y.No = 0.056, S.No = 0.013. n_Y_ = 88, n_S_ = 88.

Next we decided to uncover senescence-dependent alterations of H3K9me3-marked constitutive heterochromatin in our model system for the following reasons: First, the role of H3K9 methylation has been demonstrated in the organization of nucleolus-associated chromatin in *Drosophila* [53]. Second, while genomic redistributions of H3K4me3 and H3K27me3 histone modifications, markers for active and facultative heterochromatin respectively, were investigated in replicative senescence in IMR90 cells [26], H3K9me3-marked chromatin dynamics was analysed only in oncogene-induced senescence [24,54]. Third, H3K9me3-marked centromeric and pericentromeric satellite repeat clusters are essential components of nucleolus-associated chromatin [15], and they are subject to senescence-associated distension (see Swanson *et al*. [29] and Fig 7). Semi-quantitative immunoblot analyses were performed to monitor the overall level of H3K9me3, and the results showed that there is a strong decrease in senescent cells compared to young, proliferating ones. However, this change goes hand in hand with a somewhat less decrease in global histone H3 level. In addition, robust decrease in Lamin B1 and no detectable alteration in Lamin A/C levels were observed (Fig 8A and S11 Fig). Next, the co-localization of the strongest H3K9me3 signals with pericentromeric HSATII repeat clusters was demonstrated in 3D immuno-FISH experiments in IMR90 cells (Fig 8B). In order to reveal senescence-associated changes in the nuclear distribution of constitutive heterochromatin, quantitative immunofluorescence experiments were performed [55]. The analysis focused on the perinucleolar space and the nuclear periphery (Fig 8C), the two main sites of heterochromatin accumulation in the human nucleus. First, the fluorescence intensity of the H3K9me3 staining was measured in the respective areas and calculated as per cent of the total fluorescence intensity. The results revealed that H3K9me3 signals decrease remarkably, from 5.2% to 2.2%, at the perinucleolar space in replicative senescence (Fig 8D). In contrast, there was only a little difference between the relative fluorescence intensities of proliferating and senescent cells at the nuclear periphery (9.5% vs. 8.4%). Second, the coefficient of H3K9me3 signal variation (C.V. = standard deviation/mean of fluorescence intensity) was calculated, which gives an indication of the heterogeneity of the staining in the respective areas (Fig 8E). The results showed a more heterogeneous staining in the nucleus of senescent IMR90 cells with 0.677 vs. 0.573 C.V. values in senescent and proliferating cells, respectively. Interestingly, at the perinucleolar space the C.V. values did not differ significantly in proliferating and senescent cells (0.576 compared to 0.555), but a significant increase was detected at the periphery in senescent compared to proliferating cells (0.709 vs. 0.632). Last, the abundance of the most heterochromatic regions in the perinucleolar space and at the nuclear periphery was evaluated. Therefore, the image containing the H3K9me3 signals was segmented by thresholding the fluorescence signals at 90% of the maximal intensity. While the abundance of the 10% brightest pixels, which represent the most heterochromatic regions, did not change significantly at the nuclear periphery (15.7% in proliferating vs. 13.7% in senescent cells), it strongly decreased at the perinucleolar space (5.6% in proliferating vs. 1.3% in senescent cells) (Fig 8F). We suppose that the global loss of H3K9me3 at the perinucleolar space (Fig 8D) is mainly due to the senescence-associated distension of satellites, which results also in the selective loss of intensely stained areas (Fig 8F). In contrast, the signal heterogeneity remains largely unaltered at the perinucleolar space (Fig 8E), which could be explained with the stable nucleolus association of NADs in senescence (Fig 6 and S9 Fig). Considering that the H3K9me3 signal distribution gets heterogeneous in other nuclear regions (Fig 8E), we conclude that the nucleolus may play a functionally important role in maintaining the 3D organization of constitutive heterochromatin in replicative senescence.

In order to collect initial insights about the epigenetic regulatory network underlying the senescence-dependent alterations of nuclear architecture, gene set enrichment analyses (GSEA) were performed (S12 and S13 Figs). Comprehensive gene sets were compiled that include genes encoding ATPase and regulatory subunits of chromatin remodelling complexes (CRC), histone chaperones regulating chromatin assembly and disassembly, DNA modifying enzymes and proteins that bind modified DNA, furthermore histone modifying enzymes (S5 Table). The results revealed a global reduction of the mRNA levels of epigenetic regulators in senescence, which correlates well with the non-proliferative status of the cells. However, the transcript levels of a small number of genes, including *ERCC6*, *BAZ2A*, *KAT2B*, *SETDB2*, *EZH1*, *KDM3A* and *KDM7A*, were significantly increased. This suggests that their gene products could be actively involved in genome regulation in replicative senescence. Notably, we also observed significant up- or down-regulation of various histone modifying enzymes that use H3K9 as substrate (S12 Fig). The result suggests that this residue may act as target point of different senescence-dependent cellular signals. To validate the results of gene expression array experiments, quantitative RT-PCR was performed (S12 Fig and S6 Table). The levels of nineteen mRNA species, which showed significant alterations in the microarray analysis, were measured in proliferating and senescent cells, and two control mRNAs (*TFRC* and *SMARCA1*) were used for normalization. Seventeen targets (*ERCC6*, *BAZ2A*, *CHD4*, *ATRX*, *CHD7*, *BAZ1B*, *NPM1*, *NCL*, *NPM3*, *CHAF1A*, *DNMT1*, *DNMT3B*, *PCAF*, *HDAC2*, *PRMT5*, *SUV39H1*, *KDM2B*) were selected based on their known nucleolar function [56]. The remaining two, *PSG4* and *LMNB1*, were selected because they show strong up- and down-regulation in senescence, respectively. The qRT-PCR results largely supported the results of the microarray analysis, as only the results for *ATRX* showed clear difference in the two independent assays. Taken together, the gene expression analyses lay the foundation for the identification of chromatin regulatory mechanisms that are involved in senescence-dependent nuclear remodelling.

## Discussion

In this study we used cell fractionation and genomics analyses to discover the features of NADs in proliferating and replicative senescent human embryonic fibroblasts and to untangle their network. Our results demonstrate that NADs are predominantly heterochromatic chromosomal domains, which remain largely associated with nucleoli also in senescent cells. We show that, despite microscopically observable nuclear and nucleolar re-organization, only sub-NADs change their association with nucleoli during cellular aging. These changes clearly correlate with altered transcriptional activity. Our data suggests that transcriptional activity-dependent disassociation is characteristic also for X escaper genes [45] located on the nucleolus-associated inactive X chromosome [57,58]. The largest senescence-dependent rearrangements of the nucleolus-associated chromatin are, however, not visible on the genomic maps of NADs, because they are caused by the distension of satellite repeat clusters that are not present in genome assemblies. We show altered nucleolar association of pericentromeric and centromeric satellite repeats, which corresponds to the SADS phenotype. Notably, SADS was shown to coincide with increased transcription of satellite repeats [59]. To summarize these findings we propose a model in which active NORs, bearing active and potentially also inactive rDNA repeats [60] and additional NOR-specific sequence elements around the rDNA clusters [61], safeguard the maintenance of the NAD network in replicative senescence. At the same time, transcriptionally activated regions of specific NADs and satellite repeat clusters get disassociated from nucleoli and consequently alter the composition of nucleolus-associated chromatin. Regarding the nuclear remodelling, we demonstrate global loss and local spatial rearrangements of H3K9me3-marked heterochromatin in replicative senescence. We screened for potential epigenetic regulators of senescence-dependent nuclear reorganization also by using a GSEA approach and observed several H3K9 modifying enzymes on the list of genes with significantly altered expression level. These results support each other, and suggest a role for this histone modification in the senescence-dependent dynamics of nucleolus-associated chromatin. Remarkably, we found also *BAZ2A* among the handful of chromatin regulator genes that show elevated expression level in senescence. The BAZ2A protein is thought to be involved in silencing and constitutive heterochromatin formation at rDNA, centromeres and telomeres [62,63], and it has been recently reported that depletion of BAZ2A promotes escape from senescence [64]. This suggests a role for BAZ2A in the regulation of a subset of H3K9me3-marked chromatin domains in senescence.

Although senescence-dependent chromatin alterations have been investigated in recent studies [24–28,54], several aspects of the nuclear changes remained unclear. One of them, the dynamics of nucleolus-associated chromatin, has been addressed here. Still, the picture is far from complete, and differences in the experimental systems make direct comparisons between the different studies often difficult. For instance, SAHF formation was not prominent in our replicative senescence experimental setup, which distinguishes this study from comprehensive chromatin and nuclear architecture analyses of oncogene-induced senescence (OIS) with a characteristic SAHF phenotype [24,27,54]. Nevertheless, the changes observed in constitutive heterochromatin rearrangements in replicative senescence in our work are partially consistent with the results of OIS and other replicative senescence studies. Most importantly, the alterations of H3K9me3 patterns at the nuclear periphery are reminiscent of its previously reported reorganization in LADs [24]. This reorganization accompanies senescent-dependent Lamin B1 depletion, which was demonstrated also here. Notably, we find about 70% overlap between the genomic maps of NADs and LADs in human fibroblasts, which is in agreement with the stochastic association of several chromosomal domains with either the lamina or the nucleolus [65,66], and in general with the probabilistic model of the spatial organization of the human genome [3]. Indeed, when nucleoli are in close proximity to the lamina, several chromosomal domains may also be simultaneously associated with both nuclear compartments. We predict that NADs on chromosomes bearing an active NOR show higher contact frequencies with the nucleolus than with the lamina. Our chromosome-based NAD-LAD comparison, and a pioneering single-cell genomic analysis of LADs in KBM7 cells seem to support this idea [67]. However, this issue should be addressed by single-cell nucleolus genomics in future studies, in which the dissimilar senescence-dependent dynamics of LADs and NADs could also be more precisely addressed. We hypothesize that globally lowered contact frequencies with the nuclear periphery may accompany with increased nucleolar contact frequencies. This scenario would be consistent with the senescence-dependent destruction of the lamina and the simultaneous enlargement of nucleoli. Notably, the latter one correlates with increased ribosomal RNA precursor levels, which is due to delayed processing and acts as a senescence inducer [68]. In a next step, in nuclei undergoing SAHF formation the association probability of chromosomal domains with the nucleolus might also be reduced.

Concerning the senescence-independent role of NADs in chromosome organization, our bioinformatics analyses of high-resolution IMR90 Hi-C data [48] revealed that NAD-NAD interactions are the dominant ones in the 10–50 Mb genomic distance range and in intra-chromosome-arm contacts, whereas iNAD-iNAD interactions are more frequent over larger genomic distances. The high interaction frequency of NADs in the 10–50 Mb distance range correlates well with the current view that a compact, transcriptionally inactive nuclear compartment builds the core of chromatin domain clusters [69]. Importantly, the pattern of NADs and iNADs largely resembles that of the TADs, which connects the spatial organization of the interphase genome to the nucleolus, the centre of ribosome biogenesis. As ribosomal RNA synthesis determines nucleolar assembly and represents the primary response site of cell growth regulation, we speculate that active NORs may physically link the cell’s metabolic activity to 3D genome organization. Notably, the spatial organization of the genome can be considered as moderator of chromosomal communication [70]. The presented study and several previous observations about the role of the nucleolus in shaping genome architecture from yeast to human (reviewed in [12–14,71,72]) lead us to postulate that the nucleolus is an important factor in moderating this communication.

## Methods

### Cell culture and nucleolus isolation

Human IMR90 embryonic fibroblasts were obtained from Coriell Repositories (Cat. No. I90-79) and cultivated in DMEM (Gibco Cat. No. 21885–025 supplemented with 10% v/v Foetal Calf Serum, 100 U/mL Penicillin, 100 μg/mL Streptomycin) at 37°C in humidified, 5% CO_2_ atmosphere and regularly tested for mycoplasma contamination. Proliferating young and senescent cells were cross-linked with 1% formaldehyde and nucleoli were isolated as described [73]. Nucleolus-associated DNA was prepared from two independent experiments, both from young and senescent cells, for subsequent microarray analysis. Total genomic DNA of the same IMR90 cells served as control for one young and one senescent sample during comparative hybridization, whereas the other young and senescent samples were hybridized against non-nucleolar DNA. This DNA was collected during the nucleolus preparation and only the DNA of the last, nucleolar fraction was excluded from it. Since the depletion of nucleolus-associated DNA in the control did not alter markedly the NAD patterns, the results obtained from the two independent experiments were combined both for the young and senescent samples (S2 Fig). Quality controls of nucleolus preparations were performed as described [15].

### Cellular senescence assays

Cell populations were kept in culture for two weeks after they stopped growing. During this time the growth medium was replaced every second day. The senescence status of the IMR90 cell populations was monitored by senescence-associated beta-galactosidase staining of fixed cells (BioVision Senescence Detecion Kit), and immunofluorescence staining of the MKI67 proliferation marker protein in fixed cells by using a rabbit polyclonal antibody (Santa Cruz sc-15402).

### Computational analyses

If not stated otherwise, all computational analyses were performed in R/bioconductor using default parameters (R-project.org/bioconductor.org, version 3.3; R Core Team (2016). R: A language and environment for statistical computing. R Foundation for Statistical Computing, Vienna, Austria. URL https://www.R-project.org/). The hg19 version of the human genome served as reference. We used hg19, because most of the comparative genomics analyses were performed with datasets available in this format.

### Gene expression microarray experiments

Gene expression microarray analyses were performed using TRIzol-extracted total RNA from young, proliferating and senescent IMR90 human diploid fibroblasts. Two biological replicate experiments were performed, and the Affymetrix Human Gene 1.0 ST microarray platform was used. Labelling of the samples and hybridizations were carried out at Source BioScience. We calculated expression values using the Robust Multichip Average (RMA) algorithm. Many-to-one probesets to gene relationships were resolved by retaining the probeset with the highest variance across all arrays. In order to compare expression levels with the ones of Shah et al. (GSE36640) and Lackner et al. (E-MTAB-2086) we merged all experiments using the ‘COMBAT’ method in the bioconductor library ‘inSilicoMerging’. Hierarchical clustering on the merged set was performed on euclidean distances with the ‘complete’ method of the ‘hclust’ function in R.

### Gene set enrichment analysis

Gene sets of chromatin remodelling enzymes, histone chaperones, DNA modification enzymes and binding proteins, furthermore histone modifying enzymes were compiled based on literature search. Gene Set Enrichment Analyses (GSEA) were performed according to the instructions described on the GSEA homepage http://software.broadinstitute.org/gsea/index.jsp and in [74].

### Quantitative RT-PCR

To validate the results of the gene expression microarray experiments, RNA of young and senescent cells was prepared from three additional, independent experiments. 200 ng RNA of each sample was reverse transcribed by using 200U of MMLV-RT and random hexamers. The resulting cDNA was amplified in real-time quantitative PCR experiments using SybrGreen-intercalation-based quantification. Primer pairs used for amplification were selected using the qPrimerDepot (http://primerdepot.nci.nih.gov). Data were collected with a Rotor-Gene Q system (Qiagen) and analysed using the comparative quantitation module of the system software. The mean and standard deviation values are derived from three independent experiments analysed in triplicate quantitative PCR reactions.

### Mapping and genomics of NADs

Raw tiling array signals were subjected to quantile normalization. Nucleolar enrichment was defined as the log2-fold difference of the nucleolar signal over the background (genomic input or supernatant, respectively). Enrichment signals were smoothed per sample by sliding medians in 100 kb windows as in our previous study [15]. Subsequently, enrichments were averaged for proliferating and senescent cells, respectively. Given the bimodal nature of signal distribution, a two-state hidden Markov model (library ‘tileHMM’) was employed to classify nucleolus-associated domains with a minimum length of 10 kb (S2 Fig). The average enrichment values within each domain served as ‘NAD-scores’. Genomic features of NADs and iNADs were determined by using the UCSC Table Browser (http://genome.ucsc.edu/cgi-bin/hgTables). Jaccard similarity indices that reflect the ratio of the number of intersecting base pairs between two sets to the number of base pairs in the union of the two sets and thus serve as a similarity statistic for comparing the distribution of regions of different genomic features were calculated with bedtools’ ‘jaccard’ function (version 2.24).

### Hi-C data analysis

Raw inter- and intracromosomal contact matrices (10 kb windows, MAPQ> = 30) for the IMR90 map presented in [48] (GSE63525) were normalized using the provided normalization vectors. Each interaction was classified for interaction distance, overlap with NAD or iNAD, and chromosomal arm localization.

### 3D immuno-FISH

The 3D FISH experiments, and subsequent confocal microscopy and image analysis were essentially performed as described [15], except that series of optical sections through 3D-preserved nuclei were collected using a Leica TCS SP8 confocal system. In the localisation experiments anti-NPM1 (Santa Cruz sc-6013R or sc-56622) and different fluorescence dye-conjugated secondary antibodies, BAC clones RP11-81M8, RP11-123G19, RP11-89O2, RP11-828F4, RP11-125O21, RP5-915N17, RP11-89H10, RP11-44B13, RP11-434B14, RP11-413F20, RP11-1137G4, RP11-173M10, the ‘DJ’ cosmid clone LA14 138F10 [61], the pHr4 plasmid DNA containing the +18063/+30486 BamHI/EcoRI intergenic spacer fragment of the human rDNA (GenBank Acc. No. U13369) in pBluescript SK+ [75], furthermore 5’-biotin labelled LNA FISH probes for human HSATII, telomere and centromeric alpha-satellite detection (Exiqon) were used.

### Immunofluorescence

Cells were rinsed twice in 1xPBS and fixed in 4% paraformaldehyde in 1xPBS for 10 min. All steps were carried out at room temperature. During the last minute of fixation, few drops of 0.5% Triton X-100 in PBS were added. Cells were then washed three times in 1xPBS/0.01% Triton X-100 for 3 min, followed by 5 min in 1xPBS/0.5% Triton X-100. Finally, cells were washed twice with PBS-T (1xPBS/0.1% Tween20) for each 5 min before antibody staining. Cells were incubated with the primary antibodies (anti-NPM1: Santa Cruz sc-6013R, anti-H3K9me3: CMA318 [76], anti-H3K27me3: Active Motif 61017, anti-LMNB1: Santa Cruz sc-6216) in 4% bovine serum albumin (BSA) in PBS-T for 1h in a humidified chamber, washed in PBS-T three times for 3 min and incubated with fluorescence dye-conjugated secondary antibodies in 4% BSA in PBS-T for 1 h. After washing with PBS-T twice for 5 min, the DNA was counterstained with 50 ng/ml of DAPI in PBS-T for 5 min. Slides were rinsed in PBS-T and mounted in Vectashield (Vector). Middle optical sections of 200 nm z-stacks were selected from 8-bit CLSM (Leica TCS SP8) images based on the quality of nuclear morphology. The xy pixel size was 80.25x80.25 nm. A custom-made ImageJ script was used for semi-automated quantitative analysis of the immunofluorescence signals [55]. The following parameters were measured: i) The percentage of total nuclear fluorescence intensities of H3K9me3 within the lamina- and nucleolus-associated areas (regions of interests–ROIs), which label 240 nm distance from the edge of the DAPI and nucleolus staining, respectively, ii) The heterogeneity of the staining in the ROIs as coefficient of variation (C.V. = standard deviation/mean of fluorescence intensity [77]), iii) The percentage of the 10% brightest nuclear pixels in the two ROIs. Nucleolar number and volume measurements were performed on z-stack images acquired with a Zeiss Axiovert200 microscope, using a ‘Plan-Apochromat 63x/1.40 Oil’ objective. Whole nuclei were recorded with a z-step size of 500 nm. Nucleoli were counted and assigned to cells manually. Voxel sizes were obtained by calibrating the objective, and values were saved and transferred to FIJI/ImageJ and the volume in μm^3^ was calculated by measuring the number of object voxels using the ‘3D Objects Counter’ plug-in. The measurements were performed for both the DAPI and nucleolus staining to determine nuclear and nucleolar volumes, respectively. The resulting values were transferred to an Excel sheet and the average nucleolar volume, the total nucleolar volume per cell and the ratio of nucleolar/nuclear volume per cell was calculated. The results were displayed as bee swarm plots, on which the data points represent individual cells.

### Western blot

Whole cell extracts from the same amounts of young and proliferating cells were prepared, separated by SDS-PAGE and blotted by semidry transfer. The western blot membranes were incubated with antibodies against GAPDH (Cell Signaling, #5174), histone H3 (Abcam, ab1791), the histone modification H3K9me3 (CMA318, [76]), tubulin (Abcam ab7291), Lamin A/C (Santa Cruz, sc-20681) and Lamin B1 (Santa Cruz, sc-6216).

## Supporting information

S1 Fig Scheme and controls of nucleolus isolation. Nucleolus isolation and quality control experiments were performed as described [15,73]. Differential interference contrast (DIC) micrographs show an intact IMR90 cell (‘C’) and isolated nucleoli (‘No’). The nucleus is marked with a dashed line and blue arrows point to nucleoli. Immunoblots show robust enrichment of the nucleolar transcription factor UBTF and depletion of tubulin in the nucleolar fraction. 0.5% of each fraction of the two-step purification was loaded. ‘C’—Input (whole cell extract), ‘S1’ and ‘S2’—supernatants, ‘No’—nucleolar fraction. Quantitative PCR measurements indicate the enrichment of ribosomal DNA in nucleolus-associated DNA compared to genomic DNA (‘No’/’C’). Error bars represent the standard deviation of two nucleolus isolation experiments from young (Y1 and Y2) and replicative senescent (S1 and S2) IMR90 cells, each of which was analysed in nine technical replicate measurements (triplicate quantitative PCR reactions in three PCR runs).
(TIF)

S2 Fig Features and processing of aCGH data. (A) Scatter plot comparison of raw (unsmoothed) and smoothed (with a 100 kb window) aCGH data obtained from two independent experiments (both for young and senescent cell populations) shows the similarity of the corresponding datasets. (B) IGB snapshot view of original and smoothed aCGH data with HMM-identified NADs indicates that smoothing leads to less noise, but no remarkable changes in NAD patterns. The two upper panels show unsmoothed data from two independent experiments performed in young, proliferating IMR90 cells, the next two panels show the according smoothed data, and in the lower panel the mean values of the latter two are shown. HMM-identified NADs are shown directly below the corresponding microarray data. RefGenes on the 15Mb-65Mb region of the p-arm of HSA1 are shown at the bottom. (C) The density plots of aCGH signals indicate the bimodal nature of signal distribution, which is visible also on the IGB snapshot shown in (B).
(TIF)

S3 Fig Association of the X chromosome with the nucleolus in IMR90 cells. (A) Distribution of NADs along human sex chromosomes. NADs are indicated by red rectangles over the ideograms of the chromosomes. Note that IMR90 cells have female origin and thus one active and one inactive X chromosome. The inactive X is often completely associated with perinucleolar or peripheral heterochromatin, and therefore almost the entire chromosome is covered with NADs. The few NADs on the Y chromosome appear due to cross-hybridization on the microarrays or false assignment. (B) Visualization of the dual localization preference of the inactive X (Xi) by immunostaining is shown in the lower panel. Proliferating IMR90 cells were fixed with formaldehyde and stained with antibodies against H3K27me3, NPM1 (nucleoli) and LMNB1 (lamina). Xi appears as the strongest signal in the H3K27me3 immunostaining. A mid light-optical section of a confocal microscopy image is shown, in which most of the Xi signals are in the focal plane. H3K27me3 signals are shown in green and nucleoli/lamina signals in red on the merged image.
(TIF)

S4 Fig NAD/LAD differences on individual chromosomes. (A) Venn diagrams and Jaccard coefficients show the extent of overlap between NADs and LADs on each autosome. The IMR90 LADs from Sadaie et al. [24] were used for the analysis, because they showed the highest similarity to NADs (see Fig 2G). The sizes of overlapping and non-overlapping regions (in 10Mb) are shown above and Jaccard coefficients below the diagrams. Chromosome numbers are shown in bold in the middle of each Venn diagram. (B) The proportions of chromosomal NAD regions that do not overlap with LADs (%NAD-not-LAD = (NAD-LAD size)/(chromosome size)x100) are shown in the diagram. In the case of the acrocentrics the size of the annotated q-arm was considered as ‘chromosome size’. Note, that four of the NOR-bearing, acrocentric chromosomes (13, 14, 21, 22) are NAD-dominated. Since chromosome 15 appears as LAD-dominated in this analysis, we speculate that its NOR contains mostly inactive rDNA repeats in the IMR90 cells used in this study. (C) Integrative Genome Browser snapshots of chromosomes 17 and 22. Chromosomal regions that are covered only by LADs (LAD-NAD) or NADs (NAD-LAD) are shown above the LAD and NAD tracks as indicated. Chromosome 17 is clearly LAD-dominated, whereas chromosome 22 is NAD-dominated.
Collectively, the results shown here highlight the nucleolus-targeting potential of NORs, while showing also differences between NORs of different chromosomes. In addition, HSA19 and HSA4 are also particularly enriched in NADs, which is in good agreement with our previous observations [15]. HSA19, a chromosome localized usually internal in the nucleus, contains a large number NADs which do not appear as LADs. The ‘top NADs’ of HSA4 include the centromeric and both subtelomeric regions, which suggests tethering or anchoring of the chromosome by these regions to the nucleolus.
(TIF)

S5 Fig NAD-NAD interactions dominate in the 10–50 Mb distance range over iNAD-iNAD and heterotypic NAD-iNAD interactions. Average frequency plots of intrachromosomal NAD-NAD, iNAD-iNAD and NAD-iNAD interactions at different distances on all autosomes. Intraarm (pp, qq) and interarm (pq) interactions are shown separately as in Fig 2A. Note that the p-arms of the five acrocentric chromosomes were not (13, 14, 15 and 22) or only partially (21) analysed because they are not present in the current human genome assembly.
(TIF)

S6 Fig Correlation of Hi-C contact and NAD/iNAD patterns on individual chromosomes. Intracromosomal contact matrices of the IMR90 map from Rao et al. 2014 (GSE63525) were combined with NAD/iNAD maps of young, proliferating IMR90 cells. Diagrams on the left side show all Hi-C contact frequencies. The intensity of the blue colour correlates with the contact frequency. To aid the visualization of homotypic contacts, iNADs and NADs were masked resulting in NAD-NAD, iNAD-iNAD diagrams, respectively, which are shown on the right side. Interaction classes are illustrated in the diagrams of the second column from the left: red–NAD-NAD, green–iNAD-iNAD, yellow–mixed interactions. Note the striking similarity between long-range (>5 Mb) Hi-C contact frequency map patterns (TADs) and NAD-iNAD segmentation of the chromosomes.
(TIF)

S7 Fig Genome-wide NAD-NAD, iNAD-iNAD and NAD-iNAD interaction frequencies. Boxplots show average intraarm (pp, qq) and interarm (pq) interactions. Data points are the average interaction values of 10 kb windows matching a specific interaction class (NAD-NAD, iNAD-iNAD, mixed) and calculated for each autosome (n = 22).
(TIF)

S8 Fig Controls of replicative senescence in IMR90 cell populations. Complete growth curve of IMR90 cells shows the kinetics of the cell population growth until reaching senescence. Mean and standard deviations of triplicates of cell counting are shown. Log2 of the cell number was plotted against time. Overexpression of senescence-associated beta-galactosidase was inspected using an enzyme activity assay as shown next to the growth curve. IMR90 cells were fixed with formaldehyde and beta-galactosidase activity assay was performed. Intense blue staining marks senescent cells. The level of the proliferation marker protein MKI67 was monitored by immunofluorescence staining shown in the bottom panel. Young and senescent IMR90 cells were fixed with formaldehyde and stained for MKI67 and DAPI. Young, proliferating cells show positive MKI67 staining, whereas senescent cells are negative for MKI67. MKI67 staining is labelled in red, and DAPI staining in blue on the merged image.
(TIF)

S9 Fig High-resolution maps of NADs on autosomes in young (Y—red) and senescent (S–blue) cells. Genomic regions associated with nucleoli only in young (¬S—red) or senescent (¬Y—blue) cells are shown also as individual tracks.
(TIF)

S10 Fig Hierarchical clustering of gene expression array data demonstrates the similarity of the cell populations analysed in three different laboratories. Note the influence of the array platform on co-clustering.
(TIF)

S11 Fig Semi-quantitative immunoblots show severely decreased H3K9me3 and Lamin B1 levels, less strongly decreased H3 levels and no detectable alterations in Lamin A/C, tubulin and GAPDH levels in senescence. The same amounts of whole cell extracts of young and senescent cells were loaded as serial two-fold dilutions on SDS-PA gels and analysed on immunoblots. Cell extracts were collected from three independent experiments. Arrows label the signals related to the indicated antigens, and stars mark background signals of the previous immunodetection on the same membrane.
(TIF)

S12 Fig Gene set enrichment analysis (GSEA) outlines the epigenetic regulatory network of senescence-dependent chromatin alterations and suggests that histone H3K9 modifications are involved in this process. (**A**) GSEA of epigenetic regulators (see S5 Table for the full list) reveals frequent decrease (blue bars) in their mRNA levels in senescence. The few genes with increased mRNA levels, which are indicated in red, can be considered as active regulators of senescence. Bar graphs show log2 fold changes in mRNA levels of senescent vs. young cells. Epigenetic regulators with known nucleolar, rDNA-related activity are shown in bold, and regulatory subunits of chromatin remodelling complexes (CRC) in italic. Histone modifying enzymes that act certainly (n = 6) or possibly (n = 5) on H3K9 are labelled with dark and light grey background, respectively. (**B**) Quantitative RT-PCR validation of selected microarray data. Relative amounts of specific mRNA species in total RNA preparations from young and senescent cells were determined by quantitative RT-PCR with primer pairs listed in S6 Table. The bar graphs depict log2 fold changes in mRNA levels of senescent vs. young cells as determined using two different calibrator mRNA species in qRT-PCR experiments. The log2 fold changes measured in microarray experiments are shown next to the corresponding qRT-PCR data (see legend of the graph). Error bars represent the standard deviation of three independent biological replicate experiments, each of which was analysed in triplicate quantitative PCR reactions.
(TIF)

S13 Fig Gene set enrichment analysis (GSEA) of epigenetic regulators demonstrates frequent decrease in their mRNA levels in replicative senescence, and it reveals putative active epigenetic regulators with increased mRNA levels in senescent cells. GSEA was performed by using the GSE80447 (this study) and the GSE36640 gene expression array datasets, and custom-made gene sets. GSEA enrichment plots are shown for ‘CHROMATIN DYNAMICS’, ‘HISTONE MODIFICATION’ and ‘DNA MODIFICATION’ gene sets on the top. Full gene sets are shown on the bottom and also in S5 Table. The genes on the GSEA output images are ordered according to decreasing senescent vs. young (S/Y) expression ratios. High and low mRNA levels are marked with red and blue, respectively. Note the occasional differences between the results of the two studies (e.g. NAP1L3), indicating that the S/Y expression ratios of certain genes might be influenced by factors other than the senescence status. The Enrichment in Phenotype sections of the analysis reports are shown in tabular format on the right bottom side, below the ‘DNA MODIFICATION’ analysis data.
(TIF)

S1 Table Genomic coordinates (hg19 genome build) of IMR90 NADs (this study) and HeLa top NADs [15]. (XLSX)

S2 Table Single cell measurements of nucleolus association frequencies of twelve selected genomic regions support the results of high-throughput genomics experiments. 3D immuno-FISH analyses of NAD and inter-NAD regions (iNADs) were performed in young IMR90 cells in this and our previous study [15], and nucleolus association frequencies (% allele) were determined in 50 and 40–44 cells, respectively. The NAD score values of the regions of interest were calculated from the microarray experiments as average log2-fold difference of the nucleolar signal over the background. BAC names and their genomic coordinates (hg19 genome build) are indicated.
(XLSX)

S3 Table Expression features of 630 protein-coding genes on the inactive X chromosome as determined by Carrel and Willard [45]. Genomic coordinates (hg17 and hg19 genome builds) of the genes are also shown.
(XLSX)

S4 Table Genomic coordinates (hg19 genome build) and NAD scores of ‘Y only’ and ‘S only’ NADs and their genomic features compared to all NADs and all autosomes. (XLSX)

S5 Table Chromatin remodelling, histone chaperone, histone modifying enzyme and DNA modification gene sets compiled for GSEA. (XLSX)

S6 Table qRT-PCR primer sequences, genomic locations, GC%, Tm, and estimated cDNA and genomic amplicon sizes. (XLSX)

## References

[1] Cremer T, Cremer C. Chromosome territories, nuclear architecture and gene regulation in mammalian cells. Nat Rev Genet. 2001;2: 292–301. doi: 10.1038/35066075 11283701 10.1038/35066075

[2] Bickmore WA, van Steensel B. Genome architecture: domain organization of interphase chromosomes. Cell. Elsevier; 2013;152: 1270–1284. doi: 10.1016/j.cell.2013.02.001 23498936 10.1016/j.cell.2013.02.001

[3] Bickmore WA. The spatial organization of the human genome. Annu Rev Genomics Hum Genet. 2013;14: 67–84. doi: 10.1146/annurev-genom-091212-153515 23875797 10.1146/annurev-genom-091212-153515

[4] Dekker J, Marti-Renom MA, Mirny LA. Exploring the three-dimensional organization of genomes: interpreting chromatin interaction data. Nat Rev Genet. Nature Publishing Group; 2013;14: 390–403. doi: 10.1038/nrg3454 23657480 10.1038/nrg3454 PMC3874835

[5] Belmont AS. Large-scale chromatin organization: the good, the surprising, and the still perplexing. Curr Opin Cell Biol. 2014;26: 69–78. doi: 10.1016/j.ceb.2013.10.002 24529248 10.1016/j.ceb.2013.10.002 PMC3927141

[6] Pombo A, Dillon N. Three-dimensional genome architecture: players and mechanisms. Nat Rev Mol Cell Biol. 2015;16: 245–257. doi: 10.1038/nrm3965 25757416 10.1038/nrm3965

[7] Sexton T, Cavalli G. The Role of Chromosome Domains in Shaping the Functional Genome. Cell. Elsevier Inc; 2015;160: 1049–1059. doi: 10.1016/j.cell.2015.02.040 25768903 10.1016/j.cell.2015.02.040

[8] Bouwman BAM, de Laat W. Getting the genome in shape: the formation of loops, domains and compartments. Genome Biol. BioMed Central; 2015;16: 154 doi: 10.1186/s13059-015-0730-1 26257189 10.1186/s13059-015-0730-1 PMC4536798

[9] Jordan EG. Nucleolar nomenclature. J Cell Sci. 1984;67: 217–220. 6746773 10.1242/jcs.67.1.217

[10] Boisvert F-M, van Koningsbruggen S, Navascués J, Lamond AI. The multifunctional nucleolus. Nat Rev Mol Cell Biol. 2007;8: 574–585. doi: 10.1038/nrm2184 17519961 10.1038/nrm2184

[11] Hutten S, Westman BJ, Boisvert F-M, van Koningsbruggen S, Lamond AI. The Nucleolus. Genome Organization and Function in the Cell Nucleus. Wiley Online Library; 2011;: 279–307.

[12] Németh A, Längst G. Genome organization in and around the nucleolus. Trends Genet. 2011;27: 149–156. doi: 10.1016/j.tig.2011.01.002 21295884 10.1016/j.tig.2011.01.002

[13] Padeken J, Heun P. Nucleolus and nuclear periphery: velcro for heterochromatin. Curr Opin Cell Biol. 2014;28: 54–60. doi: 10.1016/j.ceb.2014.03.001 24690547 10.1016/j.ceb.2014.03.001

[14] Matheson TD, Kaufman PD. Grabbing the genome by the NADs. Chromosoma. 2016;125: 361–371. doi: 10.1007/s00412-015-0527-8 26174338 10.1007/s00412-015-0527-8 PMC4714962

[15] Németh A, Conesa A, Santoyo-Lopez J, Medina I, Montaner D, Péterfia B, et al Initial genomics of the human nucleolus. PLoS Genet. 2010;6: e1000889 doi: 10.1371/journal.pgen.1000889 20361057 10.1371/journal.pgen.1000889 PMC2845662

[16] van Koningsbruggen S, Gierlinski M, Schofield P, Martin D, Barton GJ, Ariyurek Y, et al High-resolution whole-genome sequencing reveals that specific chromatin domains from most human chromosomes associate with nucleoli. Mol Biol Cell. 2010;21: 3735–3748. doi: 10.1091/mbc.E10-06-0508 20826608 10.1091/mbc.E10-06-0508 PMC2965689

[17] Yusufzai TM, Tagami H, Nakatani Y, Felsenfeld G. CTCF tethers an insulator to subnuclear sites, suggesting shared insulator mechanisms across species. Mol Cell. 2004;13: 291–298. 14759373 10.1016/s1097-2765(04)00029-2

[18] Wong LH, Brettingham-Moore KH, Chan L, Quach JM, Anderson MA, Northrop EL, et al Centromere RNA is a key component for the assembly of nucleoproteins at the nucleolus and centromere. Genome Res. 2007;17: 1146–1160. doi: 10.1101/gr.6022807 17623812 10.1101/gr.6022807 PMC1933521

[19] Fedoriw AM, Starmer J, Yee D, Magnuson T. Nucleolar Association and Transcriptional Inhibition through 5S rDNA in Mammals. Dundr M, editor. PLoS Genet. 2012;8: e1002468 doi: 10.1371/journal.pgen.1002468 22275877 10.1371/journal.pgen.1002468 PMC3261910

[20] Smith CL, Matheson TD, Trombly DJ, Sun X, Campeau E, Han X, et al A separable domain of the p150 subunit of human chromatin assembly factor-1 promotes protein and chromosome associations with nucleoli. Mol Biol Cell. 2014;25: 2866–2881. doi: 10.1091/mbc.E14-05-1029 25057015 10.1091/mbc.E14-05-1029 PMC4161520

[21] López-Otín C, Blasco MA, Partridge L, Serrano M, Kroemer G. The Hallmarks of Aging. Cell. Elsevier Inc; 2013;153: 1194–1217. doi: 10.1016/j.cell.2013.05.039 23746838 10.1016/j.cell.2013.05.039 PMC3836174

[22] van Deursen JM. The role of senescent cells in ageing. Nature. 2014;509: 439–446. doi: 10.1038/nature13193 24848057 10.1038/nature13193 PMC4214092

[23] Bridger JM, Boyle S, Kill IR, Bickmore WA. Re-modelling of nuclear architecture in quiescent and senescent human fibroblasts. Curr Biol. 2000;10: 149–152. 10679329 10.1016/s0960-9822(00)00312-2

[24] Sadaie M, Salama R, Carroll T, Tomimatsu K, Chandra T, Young ARJ, et al Redistribution of the Lamin B1 genomic binding profile affects rearrangement of heterochromatic domains and SAHF formation during senescence. Genes Dev. 2013;27: 1800–1808. doi: 10.1101/gad.217281.113 23964094 10.1101/gad.217281.113 PMC3759696

[25] Shah PP, Donahue G, Otte GL, Capell BC, Nelson DM, Cao K, et al Lamin B1 depletion in senescent cells triggers large-scale changes in gene expression and the chromatin landscape. Genes Dev. 2013;27: 1787–1799. doi: 10.1101/gad.223834.113 23934658 10.1101/gad.223834.113 PMC3759695

[26] Cruickshanks HA, McBryan T, Nelson DM, VanderKraats ND, Shah PP, van Tuyn J, et al Senescent cells harbour features of the cancer epigenome. Nat Cell Biol. 2013;15: 1495–1506. doi: 10.1038/ncb2879 24270890 10.1038/ncb2879 PMC4106249

[27] Chandra T, Ewels PA, Schoenfelder S, Furlan-Magaril M, Wingett SW, Kirschner K, et al Global reorganization of the nuclear landscape in senescent cells. CellReports. 2015;10: 471–483. 10.1016/j.celrep.2014.12.055 PMC4542308 25640177

[28] Criscione SW, De Cecco M, Siranosian B, Zhang Y, Kreiling JA, Sedivy JM, et al Reorganization of chromosome architecture in replicative cellular senescence. Sci Adv. 2016;2: e1500882 doi: 10.1126/sciadv.1500882 26989773 10.1126/sciadv.1500882 PMC4788486

[29] Swanson EC, Manning B, Zhang H, Lawrence JB. Higher-order unfolding of satellite heterochromatin is a consistent and early event in cell senescence. J Cell Biol. 2013;203: 929–942. doi: 10.1083/jcb.201306073 24344186 10.1083/jcb.201306073 PMC3871423

[30] Narita M, Nũnez S, Heard E, Narita M, Lin AW, Hearn SA, et al Rb-mediated heterochromatin formation and silencing of E2F target genes during cellular senescence. Cell. 2003;113: 703–716. 12809602 10.1016/s0092-8674(03)00401-x

[31] Kosar M, Bartkova J, Hubackova S, Hodny Z, Lukas J, Bartek J. Senescence-associated heterochromatin foci are dispensable for cellular senescence, occur in a cell type- and insult-dependent manner and follow expression of p16(ink4a). Cell Cycle. 2011;10: 457–468. doi: 10.4161/cc.10.3.14707 21248468 10.4161/cc.10.3.14707

[32] Illner D, Zinner R, Handtke V, Rouquette J, Strickfaden H, Lanctôt C, et al Remodeling of nuclear architecture by the thiodioxoxpiperazine metabolite chaetocin. Exp Cell Res. 2010;316: 1662–1680. doi: 10.1016/j.yexcr.2010.03.008 20302859 10.1016/j.yexcr.2010.03.008

[33] Mehta IS, Figgitt M, Clements CS, Kill IR, Bridger JM. Alterations to nuclear architecture and genome behavior in senescent cells. Ann N Y Acad Sci. 2007;1100: 250–263. doi: 10.1196/annals.1395.027 17460187 10.1196/annals.1395.027

[34] Hein N, Sanij E, Quin J, Hannan KM, Ganley A, Hannan RD. The nucleolus and ribosomal genes in aging and senescence. Senescence. 2011.

[35] Kobayashi T. A new role of the rDNA and nucleolus in the nucleus—rDNA instability maintains genome integrity. Bioessays. 2008;30: 267–272. doi: 10.1002/bies.20723 18293366 10.1002/bies.20723

[36] Saka K, Takahashi A, Sasaki M, Kobayashi T. More than 10% of yeast genes are related to genome stability and influence cellular senescence via rDNA maintenance. Nucleic Acids Res. 2016;44: 4211–4221. doi: 10.1093/nar/gkw110 26912831 10.1093/nar/gkw110 PMC4872092

[37] Feser J, Tyler J. Chromatin structure as a mediator of aging. FEBS LETTERS. Federation of European Biochemical Societies; 2011;585: 2041–2048. doi: 10.1016/j.febslet.2010.11.016 21081125 10.1016/j.febslet.2010.11.016 PMC3988783

[38] Rai TS, Adams PD. Lessons from senescence: Chromatin maintenance in non-proliferating cells. Biochim Biophys Acta. 2012;1819: 322–331. doi: 10.1016/j.bbagrm.2011.07.014 21839870 10.1016/j.bbagrm.2011.07.014 PMC3895594

[39] Corpet A, Stucki M. Chromatin maintenance and dynamics in senescence: a spotlight on SAHF formation and the epigenome of senescent cells. Chromosoma. Springer Berlin Heidelberg; 2014;123: 423–436. doi: 10.1007/s00412-014-0469-6 24861957 10.1007/s00412-014-0469-6

[40] Benayoun BA, Pollina EA, Brunet A. Epigenetic regulation of ageing: linking environmental inputs to genomic stability. Nat Rev Mol Cell Biol. 2015;16: 593–610. doi: 10.1038/nrm4048 26373265 10.1038/nrm4048 PMC4736728

[41] Roadmap Epigenomics Consortium, Meuleman W, Yen A, Kheradpour P, Ziller MJ, Amin V, et al Integrative analysis of 111 reference human epigenomes. Nature. 2015;518: 317–330. doi: 10.1038/nature14248 25693563 10.1038/nature14248 PMC4530010

[42] Pope BD, Ryba T, Dileep V, Yue F, Wu W, Denas O, et al Topologically associating domains are stable units of replication-timing regulation. Nature. Nature Publishing Group; 2015;515: 402–405. 10.1038/nature13986 PMC4251741 25409831

[43] Politz JCR, Scalzo D, Groudine M. The redundancy of the mammalian heterochromatic compartment. Curr Opin Genet Dev. 2016;37: 1–8. doi: 10.1016/j.gde.2015.10.007 26706451 10.1016/j.gde.2015.10.007 PMC4911328

[44] Guelen L, Pagie L, Brasset E, Meuleman W, Faza MB, Talhout W, et al Domain organization of human chromosomes revealed by mapping of nuclear lamina interactions. Nature. 2008;453: 948–951. doi: 10.1038/nature06947 18463634 10.1038/nature06947

[45] Carrel L, Willard HF. X-inactivation profile reveals extensive variability in X-linked gene expression in females. Nature. 2005;434: 400–404. doi: 10.1038/nature03479 15772666 10.1038/nature03479

[46] Lieberman-Aiden E, Van Berkum NL, Williams L, Imakaev M, Ragoczy T, Telling A, et al Comprehensive Mapping of Long-Range Interactions Reveals Folding Principles of the Human Genome. Science. 2009;326: 289–293. doi: 10.1126/science.1181369 19815776 10.1126/science.1181369 PMC2858594

[47] Dixon JR, Selvaraj S, Yue F, Kim A, Li Y, Shen Y, et al Topological domains in mammalian genomes identified by analysis of chromatin interactions. Nature. 2012;485: 376–380. doi: 10.1038/nature11082 22495300 10.1038/nature11082 PMC3356448

[48] Rao SSP, Huntley MH, Durand NC, Stamenova EK, Bochkov ID, Robinson JT, et al A 3D map of the human genome at kilobase resolution reveals principles of chromatin looping. Cell. 2014;159: 1665–1680. doi: 10.1016/j.cell.2014.11.021 25497547 10.1016/j.cell.2014.11.021 PMC5635824

[49] Cremer T, Cremer M. Chromosome Territories. Cold Spring Harbor Perspectives in Biology. 2010;2: a003889–a003889. doi: 10.1101/cshperspect.a003889 20300217 10.1101/cshperspect.a003889 PMC2829961

[50] Cremer T, Cremer C, Lichter P. Recollections of a scientific journey published in human genetics: from chromosome territories to interphase cytogenetics and comparative genome hybridization. Hum Genet. 2014;133: 403–416. doi: 10.1007/s00439-014-1425-5 24504674 10.1007/s00439-014-1425-5

[51] Dixon JR, Gorkin DU, Ren B. Chromatin Domains: The Unit of Chromosome Organization. Mol Cell. Elsevier Inc; 2016;62: 668–680. doi: 10.1016/j.molcel.2016.05.018 27259200 10.1016/j.molcel.2016.05.018 PMC5371509

[52] Lackner DH, Hayashi MT, Cesare AJ, Karlseder J. A genomics approach identifies senescence-specific gene expression regulation. Aging Cell. 2014;13: 946–950. doi: 10.1111/acel.12234 24863242 10.1111/acel.12234 PMC4172521

[53] Peng JC, Karpen GH. H3K9 methylation and RNA interference regulate nucleolar organization and repeated DNA stability. Nat Cell Biol. 2007;9: 25–35. doi: 10.1038/ncb1514 17159999 10.1038/ncb1514 PMC2819265

[54] Chandra T, Kirschner K, Thuret J-Y, Pope BD, Ryba T, Newman S, et al Independence of repressive histone marks and chromatin compaction during senescent heterochromatic layer formation. Mol Cell. 2012;47: 203–214. doi: 10.1016/j.molcel.2012.06.010 22795131 10.1016/j.molcel.2012.06.010 PMC3701408

[55] Dillinger S, Németh A. Quantitative Immunofluorescence Analysis of Nucleolus-Associated Chromatin. Methods Mol Biol. New York, NY: Springer New York; 2016;1455: 59–69. doi: 10.1007/978-1-4939-3792-9_5 27576710 10.1007/978-1-4939-3792-9_5

[56] Németh A, Längst G. Chromatin Organization and the Mammalian Nucleolus. Proteins of the Nucleolus. 2013.

[57] Zhang L-F, Huynh KD, Lee JT. Perinucleolar targeting of the inactive X during S phase: evidence for a role in the maintenance of silencing. Cell. 2007;129: 693–706. doi: 10.1016/j.cell.2007.03.036 17512404 10.1016/j.cell.2007.03.036

[58] Yang F, Deng X, Ma W, Berletch JB, Rabaia N, Wei G, et al The lncRNA Firre anchors the inactive X chromosome to the nucleolus by binding CTCF and maintains H3K27me3 methylation. Genome Biol. BioMed Central; 2015;16: 52. 10.1186/s13059-015-0618-0 PMC4391730 25887447

[59] De Cecco M, Criscione SW, Peckham EJ, Hillenmeyer S, Hamm EA, Manivannan J, et al Genomes of replicatively senescent cells undergo global epigenetic changes leading to gene silencing and activation of transposable elements. Aging Cell. 2013;12: 247–256. doi: 10.1111/acel.12047 23360310 10.1111/acel.12047 PMC3618682

[60] Zillner K, Komatsu J, Filarsky K, Kalepu R, Bensimon A, Németh A. Active human nucleolar organizer regions are interspersed with inactive rDNA repeats in normal and tumor cells. Epigenomics. 2015;7: 363–378. doi: 10.2217/epi.14.93 26077426 10.2217/epi.14.93

[61] Floutsakou I, Agrawal S, Nguyen TT, Seoighe C, Ganley ARD, McStay B. The shared genomic architecture of human nucleolar organizer regions. Genome Res. 2013;23: 2003–2012. doi: 10.1101/gr.157941.113 23990606 10.1101/gr.157941.113 PMC3847771

[62] Guetg C, Lienemann P, Sirri V, Grummt I, Hernandez-Verdun D, Hottiger MO, et al The NoRC complex mediates the heterochromatin formation and stability of silent rRNA genes and centromeric repeats. EMBO J. 2010;29: 2135–2146. doi: 10.1038/emboj.2010.17 20168299 10.1038/emboj.2010.17 PMC2905252

[63] Postepska-Igielska A, Krunic D, Schmitt N, Greulich-Bode KM, Boukamp P, Grummt I. The chromatin remodelling complex NoRC safeguards genome stability by heterochromatin formation at telomeres and centromeres. EMBO Rep. 2013;14: 704–710. doi: 10.1038/embor.2013.87 23797874 10.1038/embor.2013.87 PMC3736129

[64] Yang L, Song T, Chen L, Soliman H, Chen J. Nucleolar repression facilitates initiation and maintenance of senescence. Cell Cycle. 2015;14: 3613–3623. doi: 10.1080/15384101.2015.1100777 26505814 10.1080/15384101.2015.1100777 PMC4825723

[65] Kind J, Pagie L, Ortabozkoyun H, Boyle S, de Vries SS, Janssen H, et al Single-cell dynamics of genome-nuclear lamina interactions. Cell. 2013;153: 178–192. doi: 10.1016/j.cell.2013.02.028 23523135 10.1016/j.cell.2013.02.028

[66] Kind J, van Steensel B. Stochastic genome-nuclear lamina interactions. Nucleus (Austin, Tex). 2014;5: 124–130. 10.4161/nucl.28825 PMC4049918 24717229

[67] Kind J, Pagie L, de Vries SS, Nahidiazar L, Dey SS, Bienko M, et al Genome-wide Maps of Nuclear Lamina Interactions in Single Human Cells. Cell. 2015;163: 134–147. doi: 10.1016/j.cell.2015.08.040 26365489 10.1016/j.cell.2015.08.040 PMC4583798

[68] Nishimura K, Kumazawa T, Kuroda T, Katagiri N, Tsuchiya M, Goto N, et al Perturbation of Ribosome Biogenesis Drives Cells into Senescence through 5S RNP-Mediated p53 Activation. CellReports. The Authors; 2015;10: 1310–1323. 10.1016/j.celrep.2015.01.055 25732822

[69] Cremer T, Cremer M, Hübner B, Strickfaden H, Smeets D, Popken J, et al The 4D nucleome: Evidence for a dynamic nuclear landscape based on co-aligned active and inactive nuclear compartments. FEBS LETTERS. 2015;589: 2931–2943. doi: 10.1016/j.febslet.2015.05.037 26028501 10.1016/j.febslet.2015.05.037

[70] Dekker J, Mirny L. The 3D Genome as Moderator of Chromosomal Communication. Cell. Elsevier; 2016;164: 1110–1121. doi: 10.1016/j.cell.2016.02.007 26967279 10.1016/j.cell.2016.02.007 PMC4788811

[71] Sáez-Vásquez J, Gadal O. Genome organization and function: a view from yeast and Arabidopsis. Mol Plant. 2010;3: 678–690. doi: 10.1093/mp/ssq034 20601371 10.1093/mp/ssq034

[72] Wang R, Mozziconacci J, Bancaud A, Gadal O. ScienceDirectPrinciples of chromatin organization in yeast: relevance of polymer models to describe nuclear organization and dynamics. Curr Opin Cell Biol. Elsevier Ltd; 2015;34: 54–60. doi: 10.1016/j.ceb.2015.04.004 25956973 10.1016/j.ceb.2015.04.004

[73] Sullivan GJ, Bridger JM, Cuthbert AP, Newbold RF, Bickmore WA, McStay B. Human acrocentric chromosomes with transcriptionally silent nucleolar organizer regions associate with nucleoli. EMBO J. 2001;20: 2867–2874. doi: 10.1093/emboj/20.11.2867 11387219 10.1093/emboj/20.11.2867 PMC125486

[74] Subramanian A, Tamayo P, Mootha VK, Mukherjee S, Ebert BL, Gillette MA, et al Gene set enrichment analysis: a knowledge-based approach for interpreting genome-wide expression profiles. Proc Natl Acad Sci USA. 2005;102: 15545–15550. doi: 10.1073/pnas.0506580102 16199517 10.1073/pnas.0506580102 PMC1239896

[75] Mais C, Wright JE, Prieto J-L, Raggett SL, McStay B. UBF-binding site arrays form pseudo-NORs and sequester the RNA polymerase I transcription machinery. Genes Dev. 2005;19: 50–64. doi: 10.1101/gad.310705 15598984 10.1101/gad.310705 PMC540225

[76] Hayashi-Takanaka Y, Yamagata K, Wakayama T, Stasevich TJ, Kainuma T, Tsurimoto T, et al Tracking epigenetic histone modifications in single cells using Fab-based live endogenous modification labeling. Nucleic Acids Res. 2011;39: 6475–6488. doi: 10.1093/nar/gkr343 21576221 10.1093/nar/gkr343 PMC3159477

[77] Zhang W, Li J, Suzuki K, Qu J, Wang P, Zhou J, et al Aging stem cells. A Werner syndrome stem cell model unveils heterochromatin alterations as a driver of human aging. Science. 2015;348: 1160–1163. doi: 10.1126/science.aaa1356 25931448 10.1126/science.aaa1356 PMC4494668

