# Supplementary figures and images for: Nucleolus association of chromosomal domains is largely maintained in cellular senescence despite massive nuclear reorganisation
Source: PLoS One. 2017 Jun 2;12(6):e0178821. doi: 10.1371/journal.pone.0178821 (PMC5456395; doi:10.1371/journal.pone.0178821)

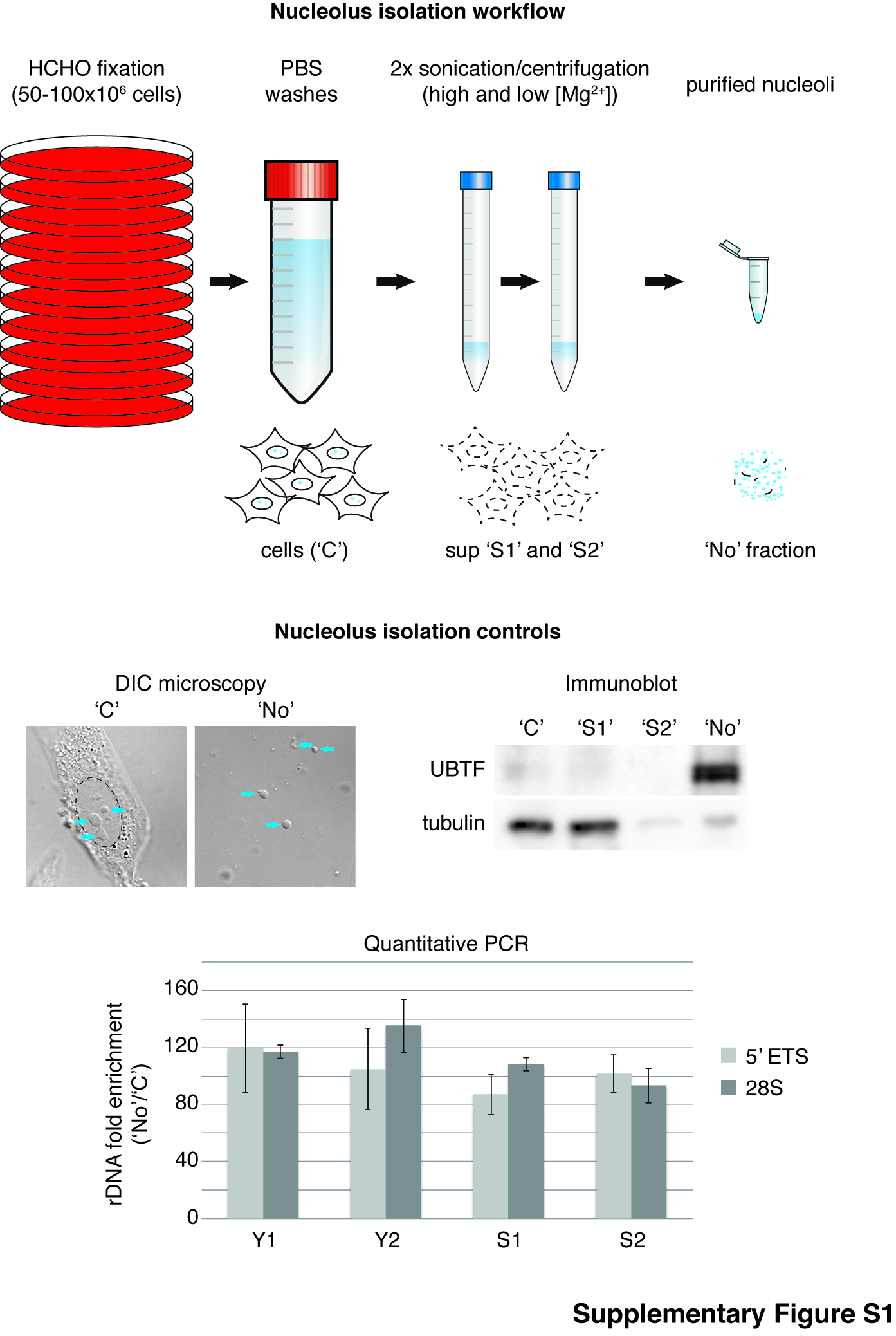

Supplement: S1 Fig — Nucleolus isolation and quality control experiments were performed as described [15,73]. Differential interference contrast (DIC) micrographs show an intact IMR90 cell (‘C’) and isolated nucleoli (‘No’). The nucleus is marked with a dashed line and blue arrows point to nucleoli. Immunoblots show robust enrichment of the nucleolar transcription factor UBTF and depletion of tubulin in the nucleolar fraction. 0.5% of each fraction of the two-step purification was loaded. ‘C’—Input (whole cell extract), ‘S1’ and ‘S2’—supernatants, ‘No’—nucleolar fraction. Quantitative PCR measurements indicate the enrichment of ribosomal DNA in nucleolus-associated DNA compared to genomic DNA (‘No’/’C’). Error bars represent the standard deviation of two nucleolus isolation experiments from young (Y1 and Y2) and replicative senescent (S1 and S2) IMR90 cells, each of which was analysed in nine technical replicate measurements (triplicate quantitative PCR reactions in three PCR runs). (TIF) [file pone.0178821.s001.tif]

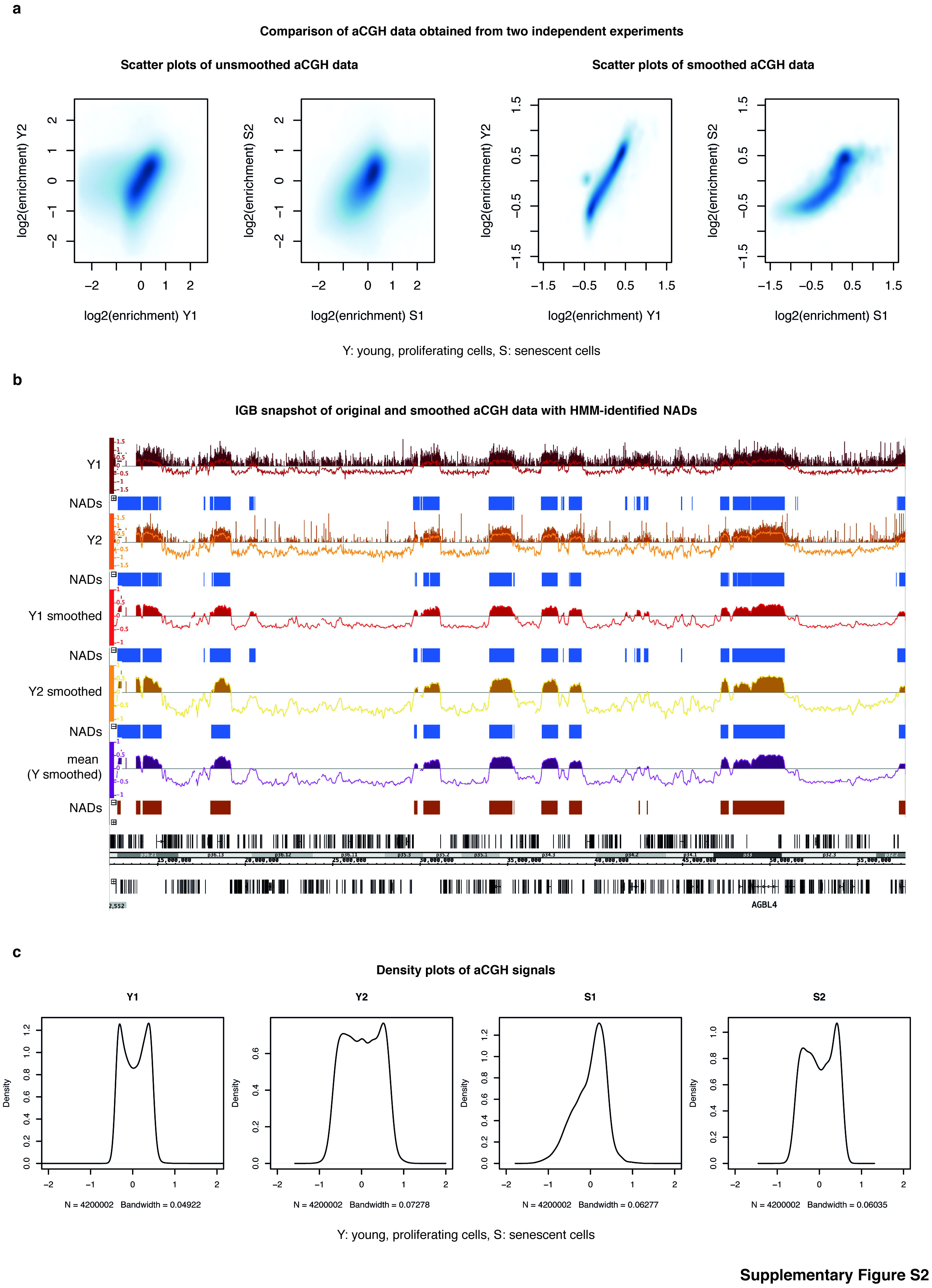

Supplement: S2 Fig — (A) Scatter plot comparison of raw (unsmoothed) and smoothed (with a 100 kb window) aCGH data obtained from two independent experiments (both for young and senescent cell populations) shows the similarity of the corresponding datasets. (B) IGB snapshot view of original and smoothed aCGH data with HMM-identified NADs indicates that smoothing leads to less noise, but no remarkable changes in NAD patterns. The two upper panels show unsmoothed data from two independent experiments performed in young, proliferating IMR90 cells, the next two panels show the according smoothed data, and in the lower panel the mean values of the latter two are shown. HMM-identified NADs are shown directly below the corresponding microarray data. RefGenes on the 15Mb-65Mb region of the p-arm of HSA1 are shown at the bottom. (C) The density plots of aCGH signals indicate the bimodal nature of signal distribution, which is visible also on the IGB snapshot shown in (B). (TIF) [file pone.0178821.s002.tif]

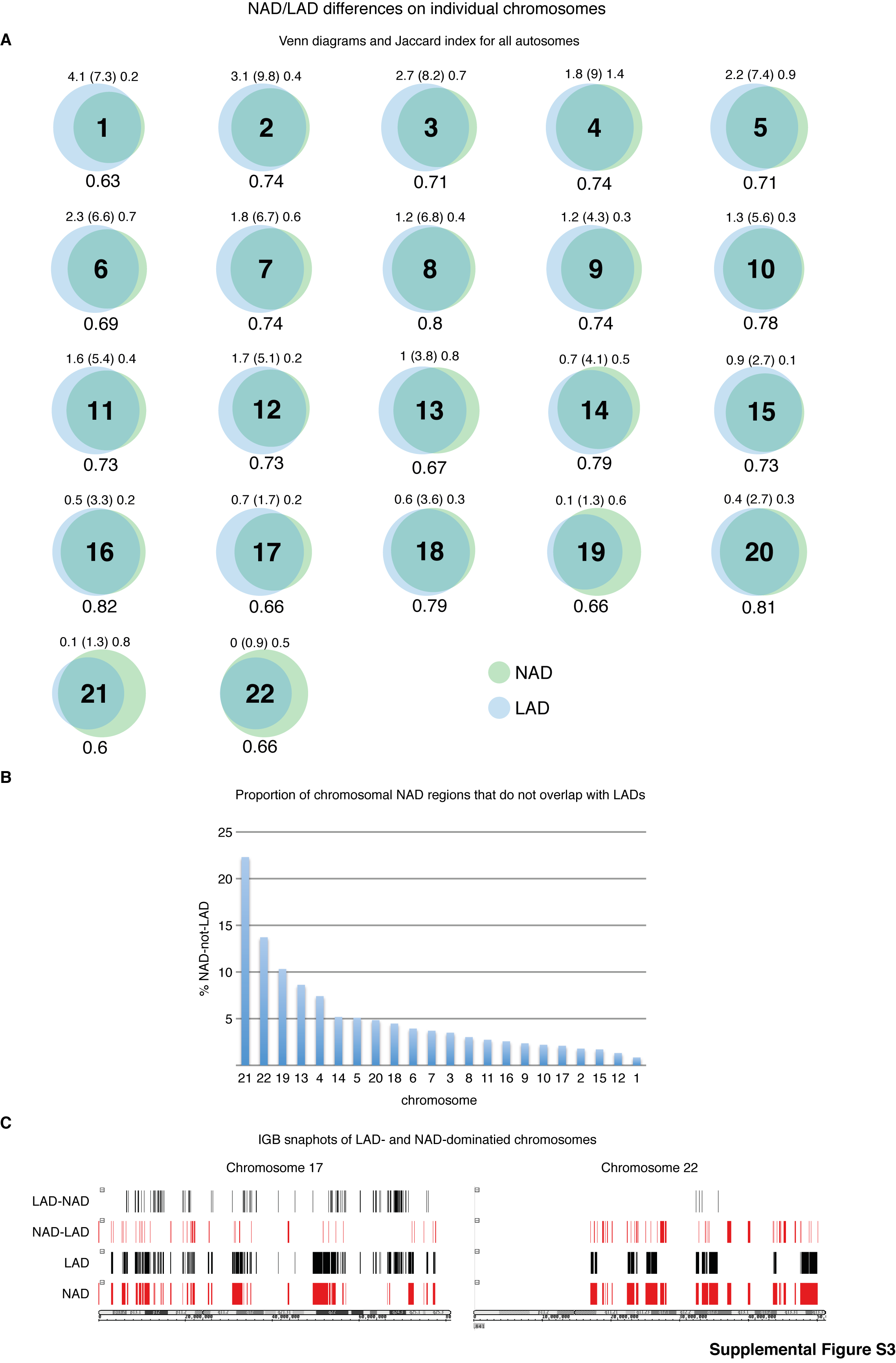

Supplement: S3 Fig — (A) Distribution of NADs along human sex chromosomes. NADs are indicated by red rectangles over the ideograms of the chromosomes. Note that IMR90 cells have female origin and thus one active and one inactive X chromosome. The inactive X is often completely associated with perinucleolar or peripheral heterochromatin, and therefore almost the entire chromosome is covered with NADs. The few NADs on the Y chromosome appear due to cross-hybridization on the microarrays or false assignment. (B) Visualization of the dual localization preference of the inactive X (Xi) by immunostaining is shown in the lower panel. Proliferating IMR90 cells were fixed with formaldehyde and stained with antibodies against H3K27me3, NPM1 (nucleoli) and LMNB1 (lamina). Xi appears as the strongest signal in the H3K27me3 immunostaining. A mid light-optical section of a confocal microscopy image is shown, in which most of the Xi signals are in the focal plane. H3K27me3 signals are shown in green and nucleoli/lamina signals in red on the merged image. (TIF) [file pone.0178821.s003.tif]

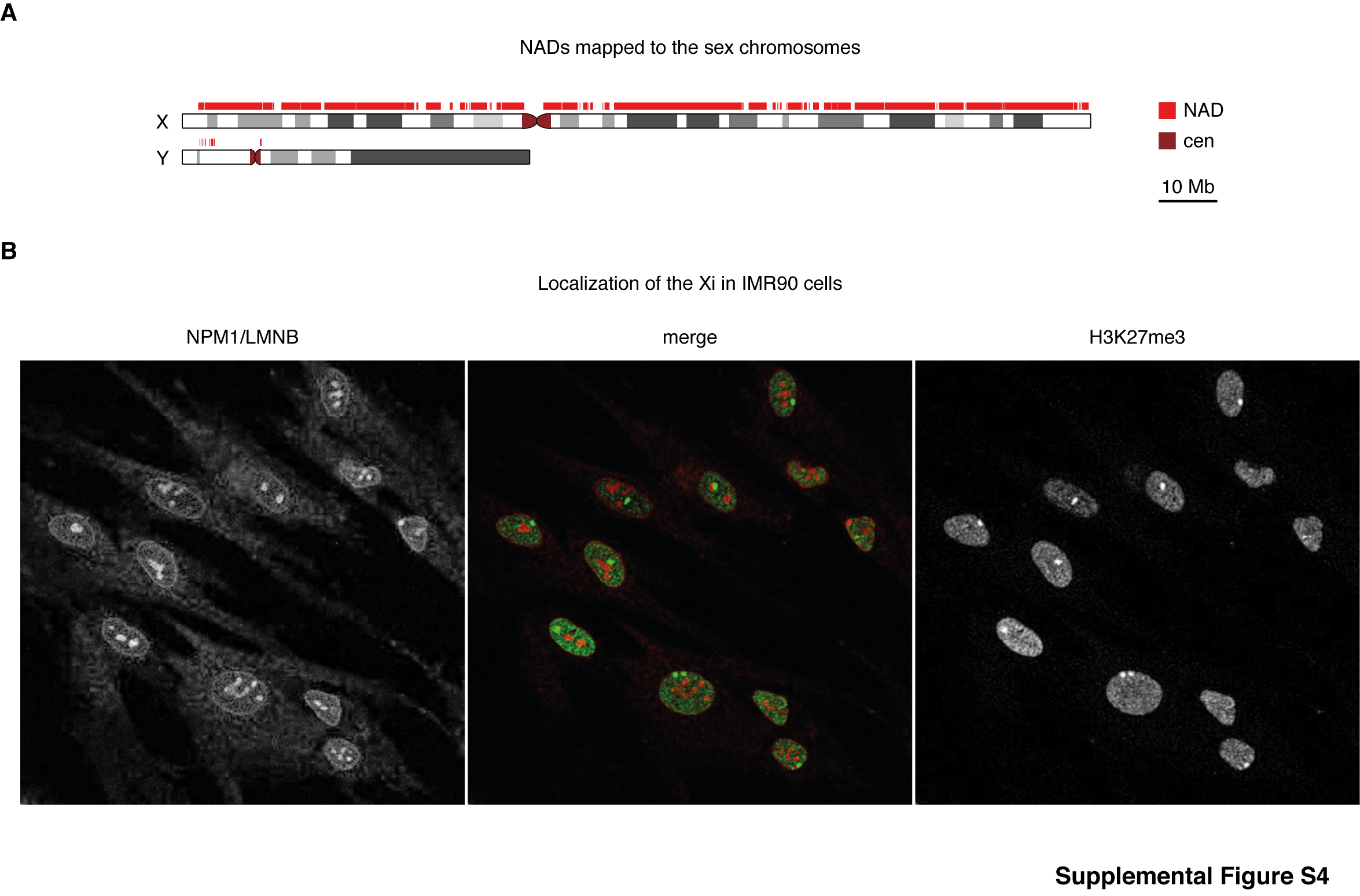

Supplement: S4 Fig — (A) Venn diagrams and Jaccard coefficients show the extent of overlap between NADs and LADs on each autosome. The IMR90 LADs from Sadaie et al. [24] were used for the analysis, because they showed the highest similarity to NADs (see Fig 2G). The sizes of overlapping and non-overlapping regions (in 10Mb) are shown above and Jaccard coefficients below the diagrams. Chromosome numbers are shown in bold in the middle of each Venn diagram. (B) The proportions of chromosomal NAD regions that do not overlap with LADs (%NAD-not-LAD = (NAD-LAD size)/(chromosome size)x100) are shown in the diagram. In the case of the acrocentrics the size of the annotated q-arm was considered as ‘chromosome size’. Note, that four of the NOR-bearing, acrocentric chromosomes (13, 14, 21, 22) are NAD-dominated. Since chromosome 15 appears as LAD-dominated in this analysis, we speculate that its NOR contains mostly inactive rDNA repeats in the IMR90 cells used in this study. (C) Integrative Genome Browser snapshots of chromosomes 17 and 22. Chromosomal regions that are covered only by LADs (LAD-NAD) or NADs (NAD-LAD) are shown above the LAD and NAD tracks as indicated. Chromosome 17 is clearly LAD-dominated, whereas chromosome 22 is NAD-dominated. Collectively, the results shown here highlight the nucleolus-targeting potential of NORs, while showing also differences between NORs of different chromosomes. In addition, HSA19 and HSA4 are also particularly enriched in NADs, which is in good agreement with our previous observations [15]. HSA19, a chromosome localized usually internal in the nucleus, contains a large number NADs which do not appear as LADs. The ‘top NADs’ of HSA4 include the centromeric and both subtelomeric regions, which suggests tethering or anchoring of the chromosome by these regions to the nucleolus. (TIF) [file pone.0178821.s004.tif]

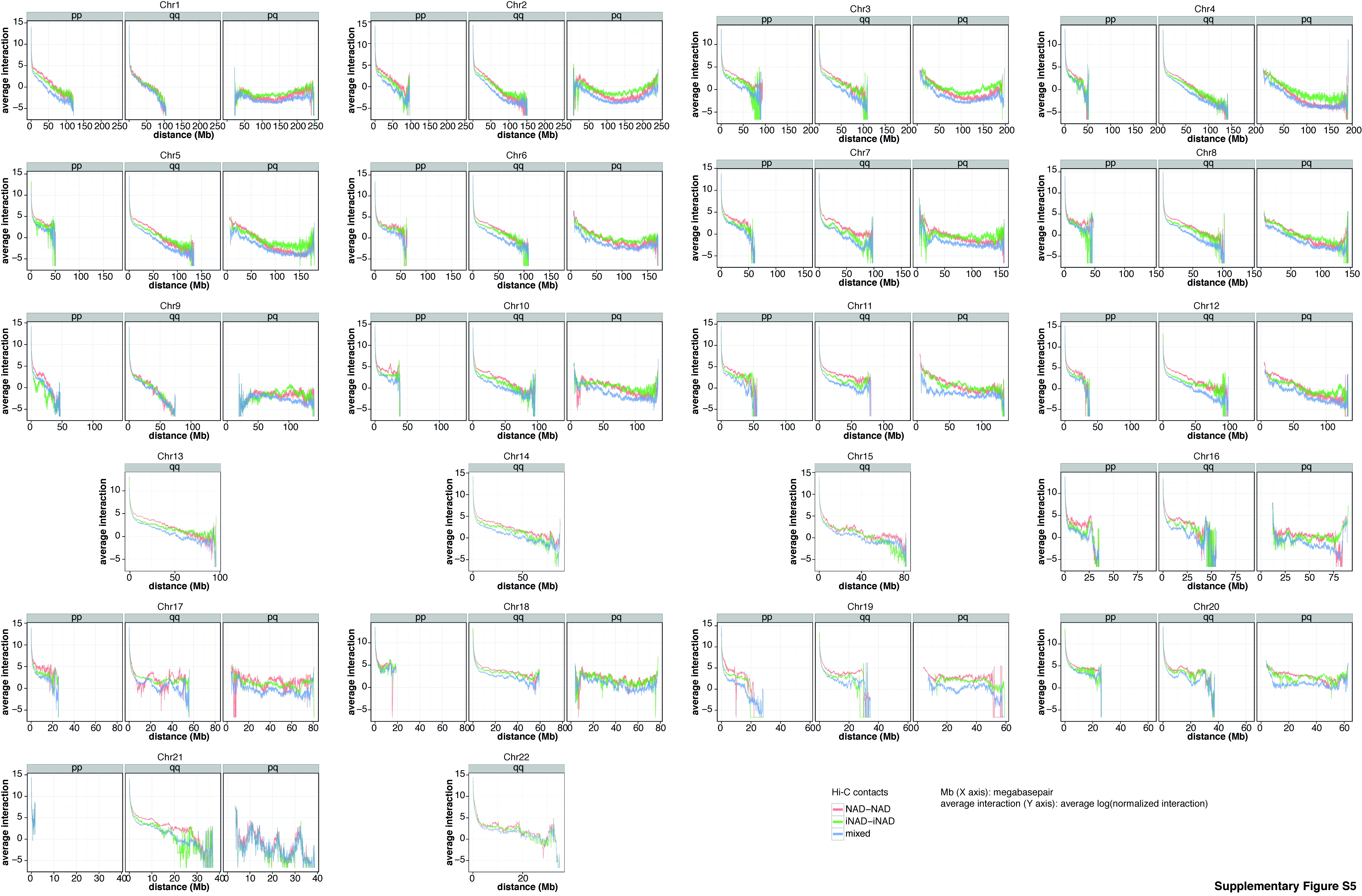

Supplement: S5 Fig — Average frequency plots of intrachromosomal NAD-NAD, iNAD-iNAD and NAD-iNAD interactions at different distances on all autosomes. Intraarm (pp, qq) and interarm (pq) interactions are shown separately as in Fig 2A. Note that the p-arms of the five acrocentric chromosomes were not (13, 14, 15 and 22) or only partially (21) analysed because they are not present in the current human genome assembly. (TIF) [file pone.0178821.s005.tif]

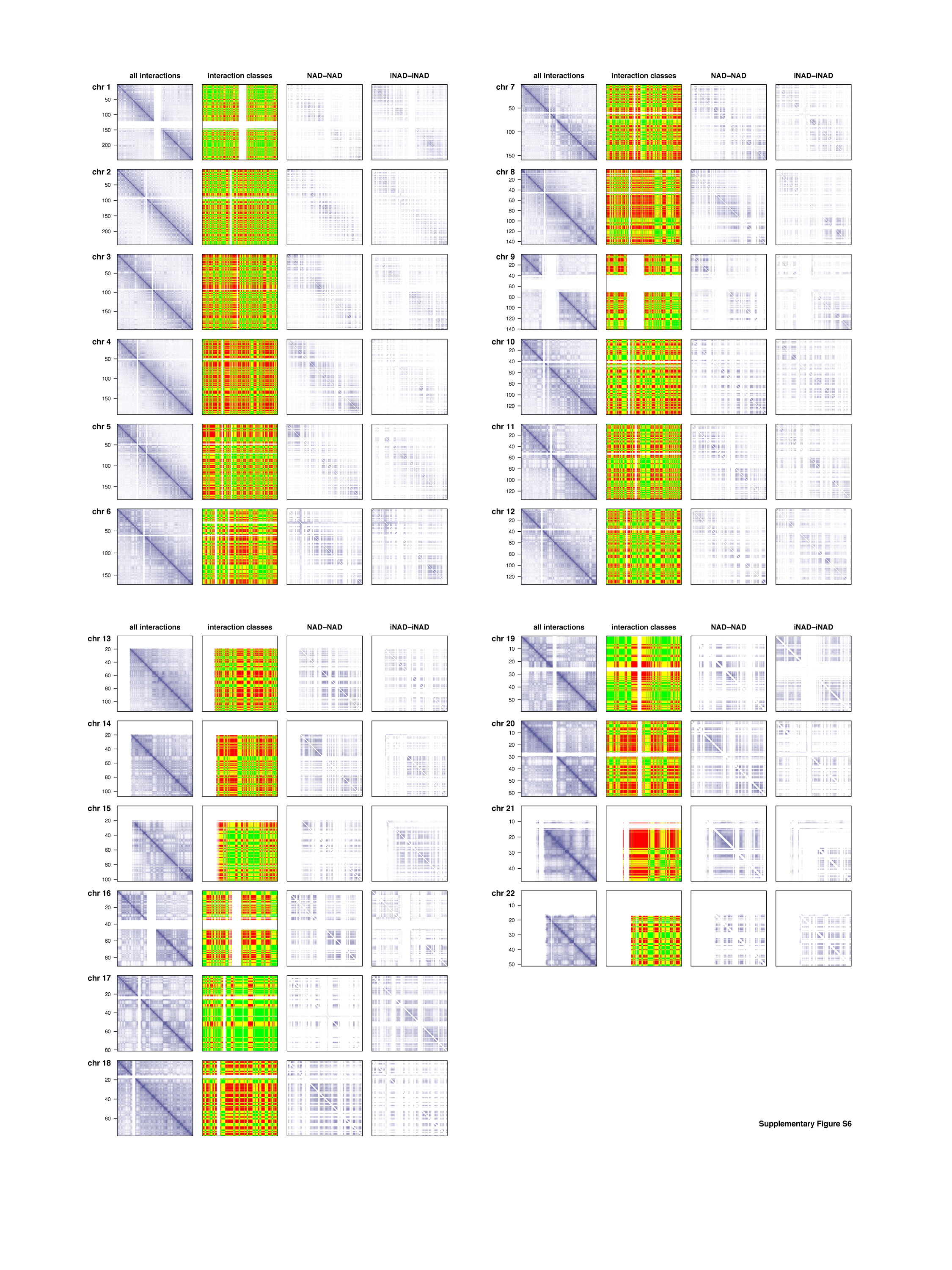

Supplement: S6 Fig — Intracromosomal contact matrices of the IMR90 map from Rao et al. 2014 (GSE63525) were combined with NAD/iNAD maps of young, proliferating IMR90 cells. Diagrams on the left side show all Hi-C contact frequencies. The intensity of the blue colour correlates with the contact frequency. To aid the visualization of homotypic contacts, iNADs and NADs were masked resulting in NAD-NAD, iNAD-iNAD diagrams, respectively, which are shown on the right side. Interaction classes are illustrated in the diagrams of the second column from the left: red–NAD-NAD, green–iNAD-iNAD, yellow–mixed interactions. Note the striking similarity between long-range (>5 Mb) Hi-C contact frequency map patterns (TADs) and NAD-iNAD segmentation of the chromosomes. (TIF) [file pone.0178821.s006.tif]

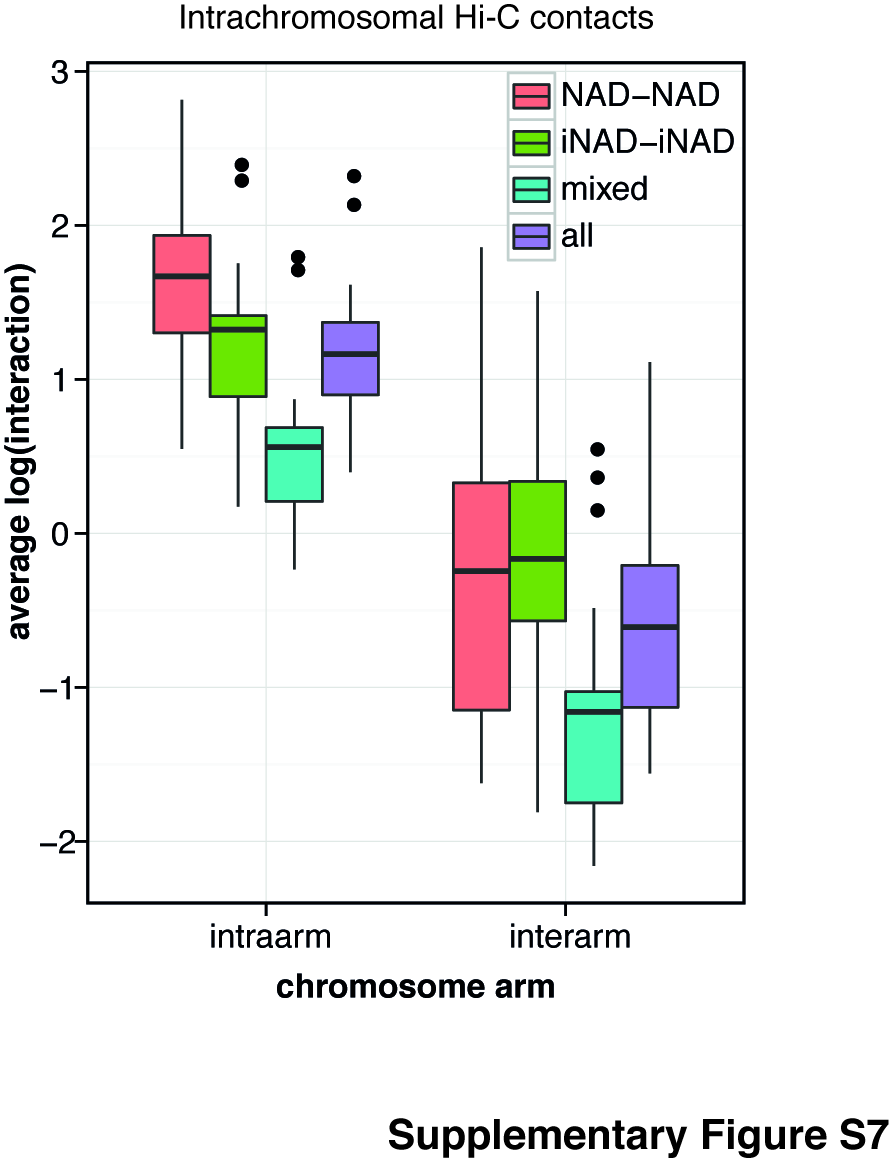

Supplement: S7 Fig — Boxplots show average intraarm (pp, qq) and interarm (pq) interactions. Data points are the average interaction values of 10 kb windows matching a specific interaction class (NAD-NAD, iNAD-iNAD, mixed) and calculated for each autosome (n = 22). (TIF) [file pone.0178821.s007.tif]

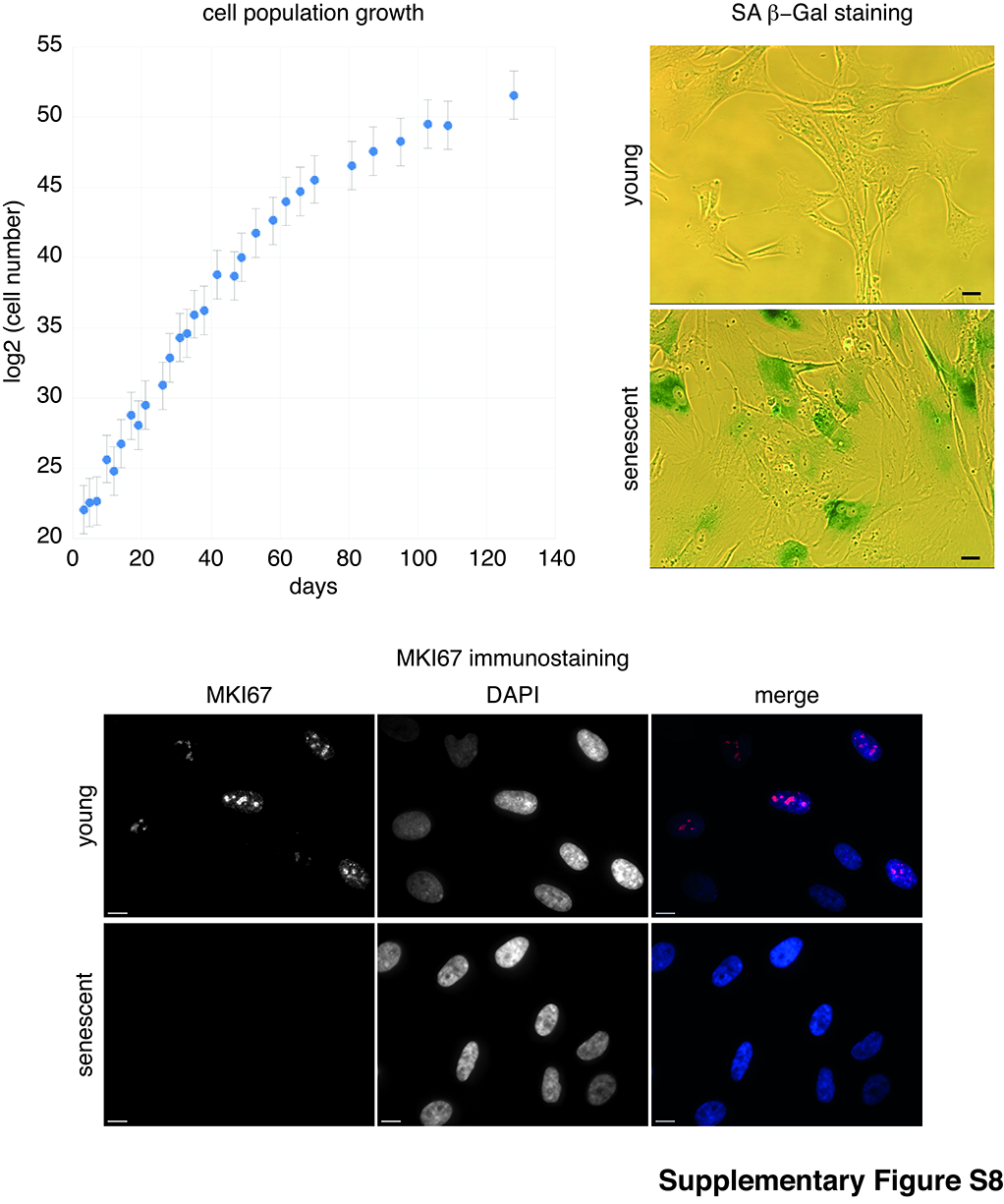

Supplement: S8 Fig — Complete growth curve of IMR90 cells shows the kinetics of the cell population growth until reaching senescence. Mean and standard deviations of triplicates of cell counting are shown. Log2 of the cell number was plotted against time. Overexpression of senescence-associated beta-galactosidase was inspected using an enzyme activity assay as shown next to the growth curve. IMR90 cells were fixed with formaldehyde and beta-galactosidase activity assay was performed. Intense blue staining marks senescent cells. The level of the proliferation marker protein MKI67 was monitored by immunofluorescence staining shown in the bottom panel. Young and senescent IMR90 cells were fixed with formaldehyde and stained for MKI67 and DAPI. Young, proliferating cells show positive MKI67 staining, whereas senescent cells are negative for MKI67. MKI67 staining is labelled in red, and DAPI staining in blue on the merged image. (TIF) [file pone.0178821.s008.tif]

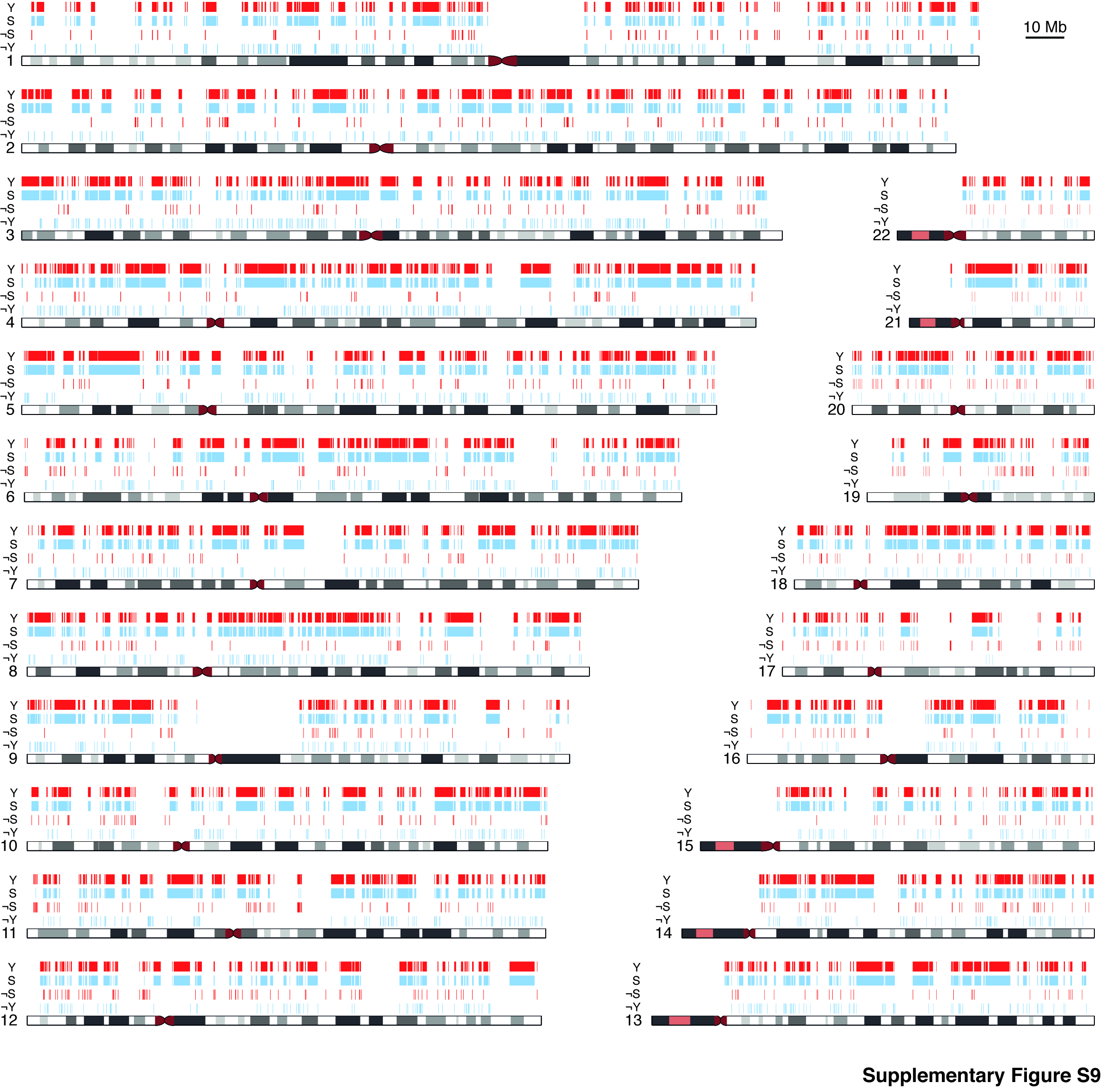

Supplement: S9 Fig — Genomic regions associated with nucleoli only in young (¬S—red) or senescent (¬Y—blue) cells are shown also as individual tracks. (TIF) [file pone.0178821.s009.tif]

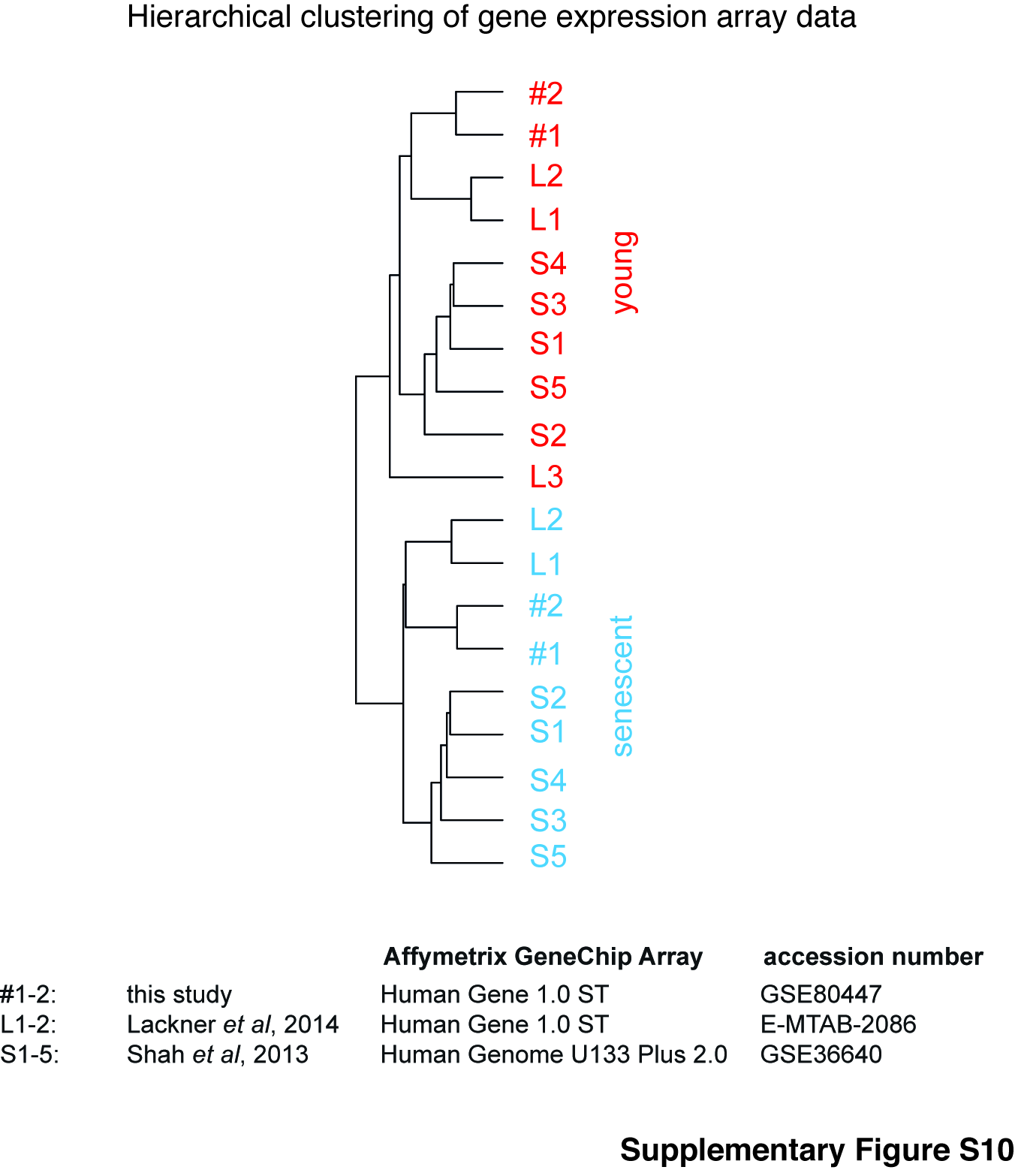

Supplement: S10 Fig — Note the influence of the array platform on co-clustering. (TIF) [file pone.0178821.s010.tif]

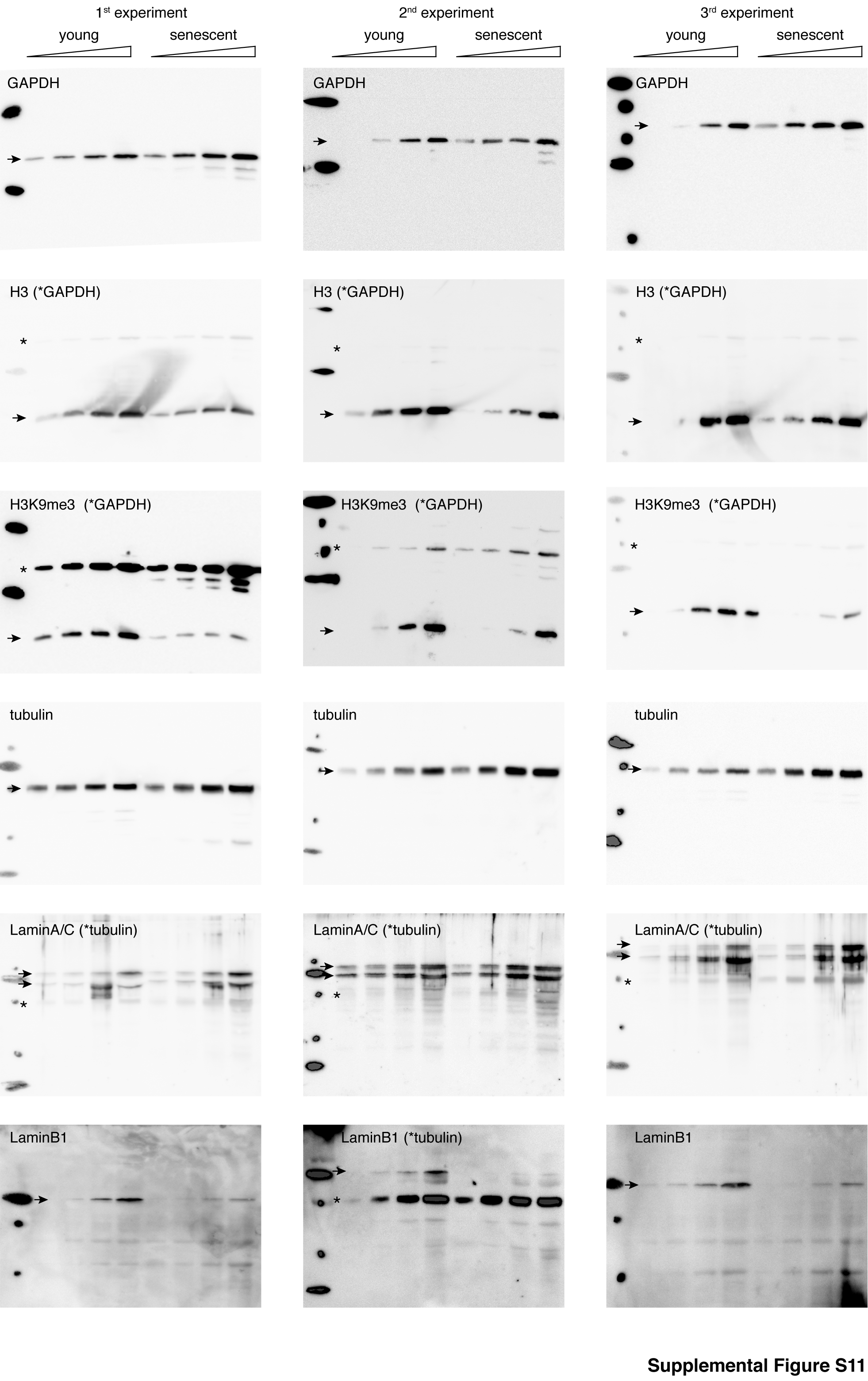

Supplement: S11 Fig — The same amounts of whole cell extracts of young and senescent cells were loaded as serial two-fold dilutions on SDS-PA gels and analysed on immunoblots. Cell extracts were collected from three independent experiments. Arrows label the signals related to the indicated antigens, and stars mark background signals of the previous immunodetection on the same membrane. (TIF) [file pone.0178821.s011.tif]

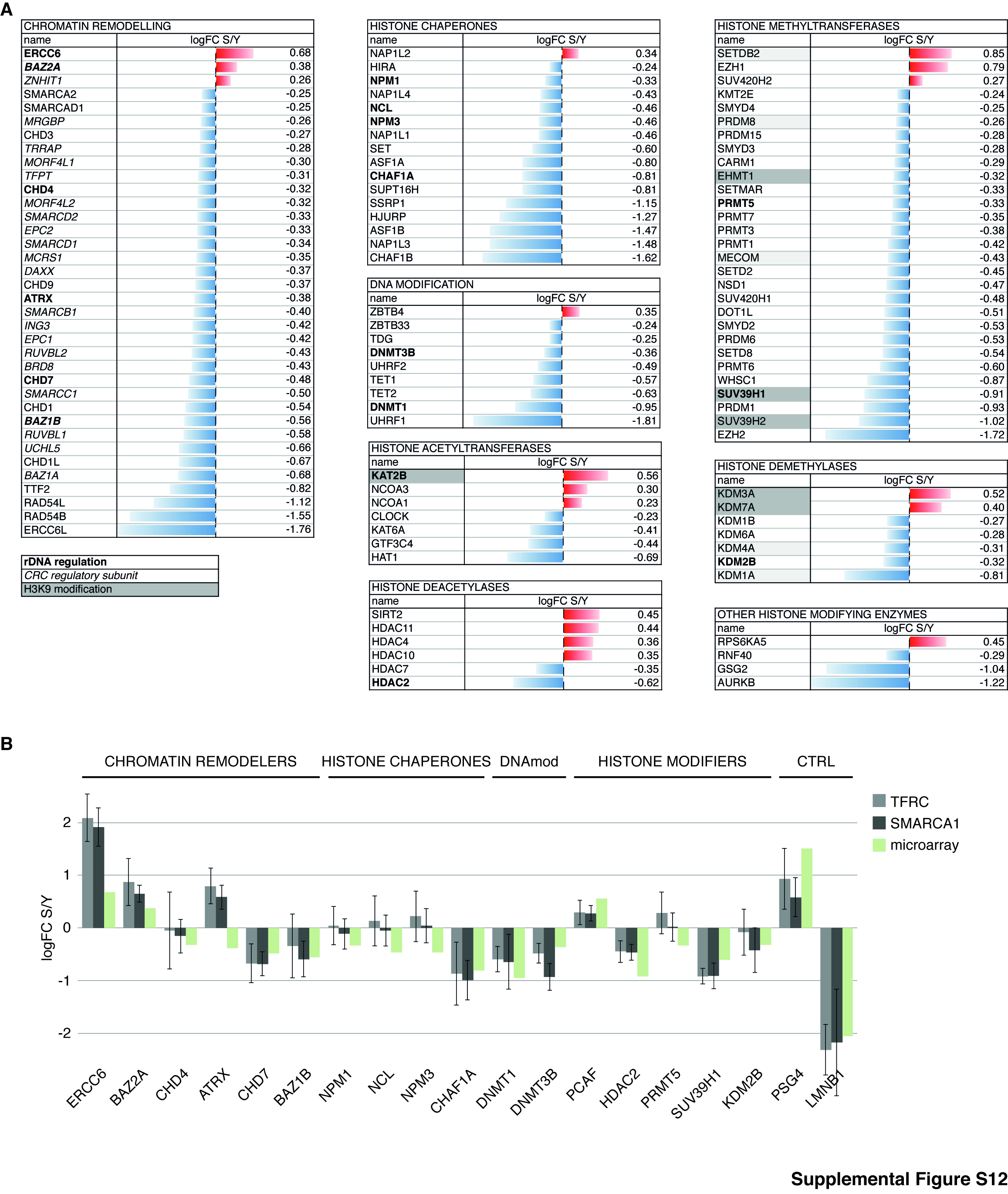

Supplement: S12 Fig — (A) GSEA of epigenetic regulators (see S5 Table for the full list) reveals frequent decrease (blue bars) in their mRNA levels in senescence. The few genes with increased mRNA levels, which are indicated in red, can be considered as active regulators of senescence. Bar graphs show log2 fold changes in mRNA levels of senescent vs. young cells. Epigenetic regulators with known nucleolar, rDNA-related activity are shown in bold, and regulatory subunits of chromatin remodelling complexes (CRC) in italic. Histone modifying enzymes that act certainly (n = 6) or possibly (n = 5) on H3K9 are labelled with dark and light grey background, respectively. (B) Quantitative RT-PCR validation of selected microarray data. Relative amounts of specific mRNA species in total RNA preparations from young and senescent cells were determined by quantitative RT-PCR with primer pairs listed in S6 Table. The bar graphs depict log2 fold changes in mRNA levels of senescent vs. young cells as determined using two different calibrator mRNA species in qRT-PCR experiments. The log2 fold changes measured in microarray experiments are shown next to the corresponding qRT-PCR data (see legend of the graph). Error bars represent the standard deviation of three independent biological replicate experiments, each of which was analysed in triplicate quantitative PCR reactions. (TIF) [file pone.0178821.s012.tif]

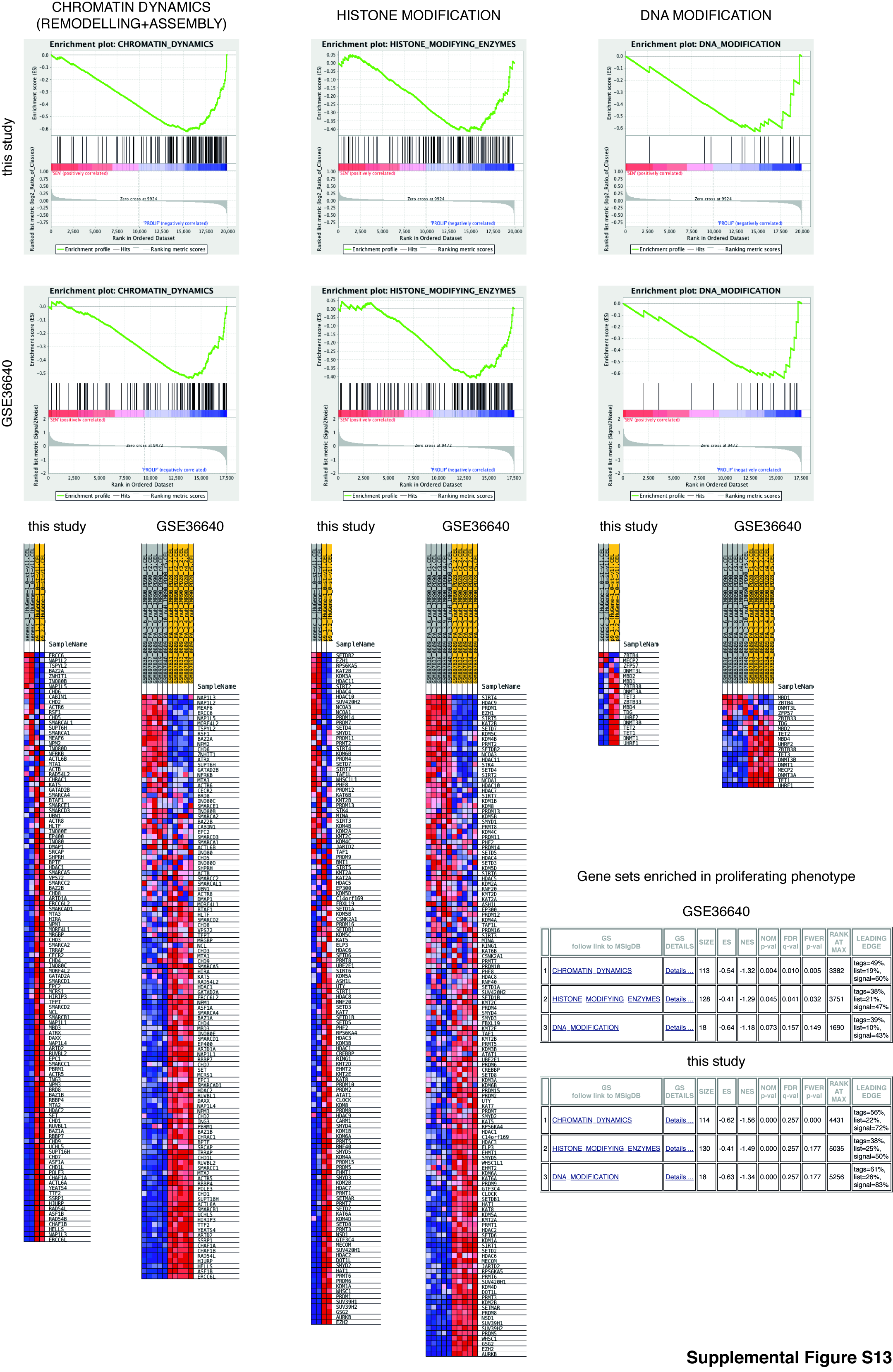

Supplement: S13 Fig — GSEA was performed by using the GSE80447 (this study) and the GSE36640 gene expression array datasets, and custom-made gene sets. GSEA enrichment plots are shown for ‘CHROMATIN DYNAMICS’, ‘HISTONE MODIFICATION’ and ‘DNA MODIFICATION’ gene sets on the top. Full gene sets are shown on the bottom and also in S5 Table. The genes on the GSEA output images are ordered according to decreasing senescent vs. young (S/Y) expression ratios. High and low mRNA levels are marked with red and blue, respectively. Note the occasional differences between the results of the two studies (e.g. NAP1L3), indicating that the S/Y expression ratios of certain genes might be influenced by factors other than the senescence status. The Enrichment in Phenotype sections of the analysis reports are shown in tabular format on the right bottom side, below the ‘DNA MODIFICATION’ analysis data. (TIF) [file pone.0178821.s013.tif]
